# Oleic Acid Biosynthesis in *Plasmodium falciparum*: Characterization of the Stearoyl-CoA Desaturase and Investigation as a Potential Therapeutic Target

**DOI:** 10.1371/journal.pone.0006889

**Published:** 2009-09-03

**Authors:** Paul Gratraud, Enlli Huws, Brie Falkard, Sophie Adjalley, David A. Fidock, Laurence Berry, William R. Jacobs, Mark S. Baird, Henri Vial, Laurent Kremer

**Affiliations:** 1 Laboratoire de Dynamique des Interactions Membranaires Normales et Pathologiques, Universités de Montpellier II et I, CNRS, UMR 5235, case 107, Montpellier, France; 2 School of Chemistry, Bangor University, Bangor, Wales; 3 Department of Microbiology, College of Physicians and Surgeons, Columbia University, New York, New York, United States of America; 4 Department of Medicine, College of Physicians and Surgeons, Columbia University, New York, New York, United States of America; 5 Howard Hughes Medical Institute, Albert Einstein College of Medicine, Bronx, New York, United States of America; 6 Department of Microbiology and Immunology, Albert Einstein College of Medicine, Bronx, New York, United States of America; 7 INSERM, DIMNP, Montpellier, France; University of Liverpool, United Kingdom

## Abstract

**Background:**

*Plasmodium falciparum* parasitization of erythrocytes causes a substantial increase in the levels of intracellular fatty acids, notably oleic acid. How parasites acquire this monounsaturated fatty acid has remained enigmatic. Here, we report on the biochemical and enzymatic characterization of stearoyl-CoA desaturase (SCD) in *P. falciparum*.

**Methodology/Principal Findings:**

Metabolic labeling experiments allowed us to demonstrate the production of oleic acid from stearic acid both in lysates of parasites incubated with [^14^C]-stearoyl-CoA and in parasite-infected erythrocytes labeled with [^14^C]-stearic acid. Optimal SCD activity was detected in schizonts, the stage of maximal membrane synthesis. This activity correlated with a late trophozoite stage-specific induction of *PFE0555w* transcripts. PFE0555w harbors a typical SCD signature. Similar to mammalian SCDs, this protein was found to be associated with the endoplasmic reticulum, as determined with PFE0555w-GFP tagged transgenic *P. falciparum*. Importantly, these parasites exhibited increased rates of stearic to oleic acid conversion, providing additional evidence that *PFE0555w* encodes the plasmodial SCD (PfSCD). These findings prompted us to assess the activity of sterculic acid analogues, known to be specific Δ9-desaturase inhibitors. Methyl sterculate inhibited the synthesis of oleic acid both with parasite lysates and infected erythrocytes, most likely by targeting PfSCD. This compound exhibited significant, rapid and irreversible antimalarial activity against asexual blood stages. This parasiticidal effect was antagonized by oleic acid.

**Conclusion/Significance:**

Our study provides evidence that parasite-mediated fatty acid modification is important for blood-stage survival and provides a new strategy to develop a novel antimalarial therapeutic based on the inhibition of PfSCD.

## Introduction

*Plasmodium falciparum* causes the most severe form of malaria and is the most widespread parasitic disease, affecting nearly 40% of the world population [1]. Up to 500 million clinical cases are reported each year and more than 1 million die annually, causing a devastating impact on the health and economic productivity of afflicted communities. Despite the availability of several licensed drugs, the emergence of multi-drug resistant strains has dramatically decreased the effectiveness of many antimalarial treatments. The development of new compounds involving novel targets and mechanisms of action is an urgent priority.

The establishment of the intraerythrocytic *P. falciparum* infection is associated with a large increase in the phospholipid, neutral lipid and lipid-associated fatty acid (FA) content in infected red blood cells (IRBCs). Intraerythrocytic proliferation largely relies on FAs derived from human serum, as shown by experiments in which parasite growth was not sustained with culture medium lacking these FAs [2], [3], [4], [5]. These studies imply that serum-derived saturated and unsaturated FAs are required for *P. falciparum* growth and that scavenging FAs from serum is critical for survival during the erythrocytic cycle [5], [6]. FA incorporation is likely to occur by passive diffusion through the membranes of the parasitized red blood cells (RBC) and the internal parasitophorous vacuole [7]. In contrast to most other organisms, where both *de novo* FA biosynthesis and modification are important for membrane homeostasis, *P. falciparum* reportedly has only minimal capacity to elongate FA during intraerythrocytic infection [7].

Sequencing of various plasmodial genomes has revealed members of the type II fatty acid (FAS-II) biosynthetic machinery, suggesting that *P. falciparum* can synthesize FAs *de novo* from acetate [8]. An earlier biochemical study reported evidence that this pathway is responsible for the synthesis of short-chain FAs and that its inhibition, by the FAS-II inhibitor triclosan, was lethal for the parasite [9]. This led to the idea that *P. falciparum* satisfies its FA requirement by scavenging free FAs present in the serum or released by the action of lipases from the lipids, and also by catalyzing *de novo* synthesis by FAS-II. Recent work, however, suggests that the FAS-II pathway is active only in liver stages and is non-essential for asexual blood stage parasites [10], [11]. These findings imply that asexual blood stages rely solely on import of host FAs. Whether these FAs can be modified during intraerythrocytic growth, including via mechanisms of desaturation, has until now not been investigated.

Earlier reports provided evidence that the intraerythrocytic *P. falciparum* parasite can salvage free FAs from the external milieu and incorporate them into its lipids without any structural modification [5], [12]. However, the presence of a putative stearoyl-CoA Δ9-desaturase (SCD), as well as several acyl-CoA synthetases, suggests that *P. falciparum* is capable of also modifying host FAs [5]. SCD is an enzyme responsible for the biosynthesis of monounsaturated FAs from saturated FAs. This iron-dependent enzyme catalyzes the insertion of a *cis* double bond at the Δ9 position of fatty acyl-CoAs with NADPH as a cofactor [13]. Several SCDs have been identified and characterized in mammals, including humans, where they have been implicated in conditions of diabetes or obesity [14]. Recently, there has been a surge of interest in the use of SCDs as chemotherapeutic targets for the treatment of various diseases, including metabolic disorders and infectious diseases. This is exemplified by the fact that the *Mycobacterium tuberculosis* SCD (termed DesA3) has been shown to be a target of the antitubercular drug isoxyl [15].

In the present study, we have undertaken a biochemical and enzymatic analysis of the putative *P. falciparum* desaturase, predicted to be required for oleic acid (C_18:1_) biosynthesis. The detection of Δ9-desaturase activity led us to investigate the effect of a specific Δ9-desaturase inhibitor on parasite viability. We conclude by discussing the potential of this class of inhibitors and the data that support PfSCD as a potential target for antimalarial intervention.

## Methods

### Parasite strains and cultures

The *P. falciparum* 3D7 strain was used for metabolic labeling and drug susceptibility testing. Using the Petri dish candle-jar method, parasites were cultured *in vitro* in complete medium, i.e. RPMI-1640 medium supplemented with 25 mM Hepes (Gibco) and 10% AB^+^ human serum [16]. The human red blood cells and sera were provided by the local Blood Center in Montpellier (France), in accordance with French legislation. The concentration of human serum in the medium was lowered to 4% for biochemical assays and drug susceptibility testing. In transfected parasites, human serum was replaced by 0.5% Albumax I (Gibco). Dd2^attB^ and 3D7^attB^ strains were grown in complete medium containing 2.5 nM WR99210 (Jacobus Pharmaceutical Co.) [17]. Synchronization of parasite cultures for stage-specific analyses was performed using 5% sorbitol [18].

### Molecular cloning, DNA constructs and *E. coli* strains

All PCR experiments were performed using the Phusion DNA Polymerase (Finnzymes). Restriction enzymes were purchased from New England Biolabs. The *Plasmodium*-specific pLN-ENR-GFP and pINT plasmids were described previously [17]. *E. coli* Top10 cells were from Invitrogen, whereas XL10 Gold and BL21(DE3) were from Stratagene.

### Generation of PfSCD-GFP transgenic parasites

Overexpression of a *Pfscd-gfp* transgene in *P. falciparum* was performed using the *attB* recombination system recently developed by Nkrumah *et al.* [17]. This system enables site-specific, stable integration of a gene of interest in Dd2^attB^ and 3D7^attB^ strains, which contain a mycobacterial *attB* recombination site inserted into the non-essential *cg6* locus. Recombination requires transfection with two plasmids: pINT that expresses the mycobacteriophage Bxb1 integrase and a neomycin selectable marker conferring resistance to G418, and pLN-ENR-GFP that permits expression of a gene of interest fused to *gfp* and that contains the mycobacterial *attP* recombination sequence as well as a blasticidin resistance cassette. Transgene expression is driven by the calmodulin promoter. We also generated a transgenic 3D7 line that replicated pLN-SCD-GFP as episomal copies.

The full-length 3 kb *Pfscd* gene was amplified from purified *P. falciparum* 3D7 genomic DNA using primers 5′-ACC TAG GTG ATA AAT GAT AGA AAT GAT CTT AAG TTA TG-3′ (*Avr*II site underlined) and 5′- ACG TAC GTA AGA ATT CCT TTA GTA CGT CC-3′ (*Bsi*WI site underlined). DNA amplification was performed using the following conditions: denaturation (94°C, 45 sec), annealing (42°C, 45 sec), polymerization (62°C, 3 min). This DNA fragment was cloned into pLN-ENR-GFP, replacing the DNA fragment encoding PfFabI (also known as PfENR), to yield pLN-SCD-GFP.

Synchronized ring stage Dd2^attB^ and 3D7^attB^ parasites (at 5–8% parasitemia) were electroporated with 75 µg of pLN-SCD-GFP and 50 µg of pINT [19]. Transfectants were selected in complete medium containing 2.5 µg/ml blasticidin (BSD, Sigma) and 125 µg/ml G418 (Invitrogen) until the emergence of transgenic parasites. Genomic DNAs from transgenic parasites and parental strains were recovered using the QIAamp DNA Blood Mini Kit, and site-specific integration of the cassette was confirmed with PCR. The following primer pairs were used: p1 (5′-ATG AAC AAA TAC ATA AGA GCG CC-3′) plus p2 (5′-ATG CAT GCC AAG CCT TTG TCT AAG-3′), and p3 (5′-ACC TAG GTG ATA AAT GAA TAT AAA TTT ATT CAA AAG AAT G-3′) plus p4 (5′-CCT TCA CCC TCT CCA CTG ACA G-3′). For episomal expression, parasites were transfected and maintained in culture under BSD selection.

### Live Imaging

Live Dd2^attB^::Pfscd-gfp infected erythrocytes were observed in PBS (137 mM NaCl, 2.7 mM KCl, 10 mM Na_2_HPO_4_, 2 mM KH_2_PO_4_), or HBSS (Gibco) on a Zeiss Axiovert 200 M epifluorescence microscope. The endoplasmic reticulum (ER) and the mitochondria were stained respectively with 0,5 µM ER Tracker Red or 20 nM MitoTracker Red (both from Molecular Probes) in HBSS supplemented with Ca^2+^ and Mg^2+^ (product reference 14025, Gibco) for 30 min at 37°C in a CO_2_ incubator. The nuclei were stained with cell permeant Hoechst H33342 (Sigma) for 5 min at room temperature (RT).

### Immunofluorescence assays

PfSCD-GFP infected RBC were fixed in 4% paraformaldehyde containing 0.0075% glutaraldehyde (both from EMS sciences) in PBS overnight at 4°C [20]. Cells were then quenched with PBS containing 0.1 M glycine for 15 min at RT, and then permeabilized with 0.1% Triton X-100 in PBS for 10 min at RT. IRBC were resuspended in 1% fetal calf serum (FCS) in PBS and incubated with the primary antibodies for 1 h at RT. After 3 washes in 1% FCS, cells were incubated with the secondary antibodies (1 h, RT), washed and layered on poly-L-Lysine coated immunofluorescence slides and mounted with Vectashield containing DAPI (Vector laboratories). All antibodies were diluted in 1% FCS: rat anti-PfBiP (1/5000 dilution) and rat anti-PfERD2 (1/1000 dilution) were from the MR4, purified rabbit anti-PfDisulfide Isomerase (1/500 dilution) was a gift from Pr P. Grellier, whereas rabbit anti-ACP (1/1000) was kindly provided by Pr. G. McFadden. Secondary antibodies, anti-rat or anti-rabbit Alexa fluor 594 nm (Molecular probes) were used at a 1/2000 dilution. Samples were observed on a Zeiss Axioimager epifluorescence microscope equipped with an Apotome for optical sectioning, using a 100X or a 63X apochromat 1.4 objective and differential interference contrast (DIC) for transmitted light. Images were processed with Axiovision, or ImageJ softwares. Image acquisition and image analysis were performed on workstations of the Montpellier RIO Imaging facility of MRI-DBS.

### Quantitative RT-PCR on time course samples *P. falciparum* mRNA

Total RNA was extracted from tightly synchronized *P. falciparum* 106/1 parasites during a 48 hrs growth period in a bioreactor (Applikon). RNA was purified as described in [21]. Contaminating DNA was removed following RQ1 DNase treatment (Promega). Double stranded cDNAs were synthesized using Superscript III Reverse Transcriptase (Invitrogen). Quantitative PCR was performed using SYBRGreen (Invitrogen) as a fluorescent probe in an Opticon2 (BioRad). *Pfscd* was amplified using the forward primer 5′-GGG ATT TAA AAT GTG CAA AGA AT-3′ and the reverse primer 5′-GCA TTG TGT AAT GAT TAT TTA AAT A-3′. The *Pfactin* gene, used as a reference, was amplified using the forward primer 5′-AGC AGC AGG AAT CCA CAC A-3′ and the reverse primer 5′-TGA TGG TGC AAG GGT TGT AA-3′. MJ Research Opticon Software (BioRad) was used to analyze the data. *Pfscd* and *Pfactin* copy numbers were determined by the absolute quantification method using a standard set of reactions of *P. falciparum* 106/1 genomic DNA of known copy number.

### Conversion of stearic acid into oleic acid in *P. falciparum*

Desaturase activity was determined at successive developmental stages of a synchronized *in vitro P. falciparum* culture. The assay was developed in 24-well plates containing IRBCs at 5% hematocrit and 5% parasitemia in a final volume of 1 ml. IRBCs were labeled for 4 hrs at 37°C with 1 µCi [^14^C]-stearic acid (C_18:0_; 56 mCi/mmol, Sigma; corresponding to a final concentration of 18 µM). Parasites were then washed once with cold PBS, transferred into glass tubes, and subjected to alkaline hydrolysis using 2 ml of 15% aqueous tetrabutylammonium hydroxide (TBAH, Sigma) overnight at 100°C. This step was followed by the addition of 2 ml of water, 4 ml of dichloromethane and 300 µl of methyl iodide, and tubes were agitated for 1 hr at RT. The upper aqueous phase was discarded while the lower organic phase was washed twice with water and dried by evaporation. Fatty acid methyl esters (FAMEs) were dissolved in diethyl ether and evaporated. The final residue was then dissolved in 200 µl of CH_2_Cl_2_. Equal counts (or equal volumes) of the resulting solution were subjected to 10% silver nitrate-impregnated thin-layer chromatography (TLC) using silica gel 1D plates (5735 silica gel 60F254; Merck). Labeled FAs were resolved in petroleum ether/diethyl ether (17/3, v/v) and autoradiograms were obtained by exposing the plate to a Kodak Biomax MR film to reveal [^14^C]-labeled FAMEs. Silver nitrate was used to separate saturated from unsaturated FAs, as the latter were retarded during migration [22]. Spots corresponding to the oleic acid methyl esters (OAMEs) were identified using a radiolabeled standard, then scraped, resuspended in scintillation liquid (UltimaGold, Perkin-Elmer) and quantified using a Beckman Coulter LS 6500 Scintillation Counter.

Substrate specificity was investigated as described above, except that parasites were labeled for 4 hrs at 37°C with either 1 µCi [^14^C]-stearic acid or 1 µCi [^14^C]-palmitic acid (56 mCi/mmol, Sigma) (each final concentration was 18 µM).

Inhibition of oleic acid synthesis in schizonts was investigated as described above, except that parasites were pre-incubated with methyl ester of cyclopropene fatty acid for 1 hr prior to the addition of [^14^C]-stearic acid (18 µM final concentration) and then incubated a further 4 hrs at 37°C.

### Cyclopropene fatty acids

Methyl esters of cyclopropene fatty acids were prepared from 1,1,2-tribromo-2-octylcyclopropane by a slight modification of the methods previously described [23], [24]. Experimental details are presented in Methods S1.

### *In vitro* stearoyl-CoA desaturase assay

Schizont-IRBCS (40 hrs post-synchronization) were harvested by centrifugation, treated with 0.05% saponin, washed once with RPMI 1640+ Hepes, and resuspended in 0.1 M potassium phosphate buffer pH 7.2 supplemented with an antiprotease cocktail (Roche Diagnostics). Schizonts were then sonicated for 30 sec and their protein content quantified using the BCA Protein Assay Reagent kit (Interchim). Cell-free desaturase activity was assayed as described previously [25], with limited modifications. Lysates were incubated with 2 mM NADPH and [^14^C]-stearoyl-CoA (56 mCi/mmol American Radiolabeled Chemicals) in a final volume of 100 µl for 5 min at 37°C. The reaction was stopped by adding 2 ml of TBAH. FAMEs were extracted, resolved by silver nitrate TLC, exposed to a film and oleic acid was quantified as described above.

In inhibition assays, schizont lysates were pre-incubated with increasing concentrations of the methyl ester of the cyclopropene fatty acid for 10 min at 37°C prior to the addition of 2 mM NADPH and 20 µM [^14^C]-stearoyl-CoA. Samples were then processed as described above.

NADPH and NADH dependency was investigated in an *in vitro* assay that used parasite membranes instead of crude lysates. Membranes (equivalent to 350 µg of proteins) were recovered by ultracentrifugation of the total extract at 100, 000×*g* for 20 min at 4°C, washed twice with potassium phosphate buffer and assayed for desaturase activity. After a 10 min pre-incubation at 37°C, 20 µM [^14^C]-stearoyl-CoA was added to the mixture in the absence or presence of either 2 mM NADPH or NADH. Samples were then processed as described above.

### Lipid analysis

Lipid composition was investigated with or without treatment with methyl sterculate (MeSter) as follows. Total lipids were extracted using 3 ml of a chloroform:methanol (2∶1, v/v) mixture [26]. After 3 hrs of incubation, 550 µl of 0.1 M KCl was added and tubes were mixed gently for another 1 hr. The upper aqueous phase was discarded and the organic phase was washed twice with a chloroform:methanol:water (3∶48∶47, v/v) mixture, then dried and resuspended in chloroform:methanol (2∶1, v/v). Equal amounts of radioactivity for each sample were loaded onto TLC plates. Phospholipids were resolved using chloroform:methanol:acetic acid:0.1 M Na borate (75∶4512∶4.5, v/v/v/v) whereas neutral lipids were separated with heptane:diisopropyl oxide:acetic acid (60∶40∶3, v/v/v) [3].

### Drug susceptibility testing to methyl-sterculate (MeSter) and its analogs

The effect of MeSter and its structural analogs on *P. falciparum* growth was determined using a modified Desjardins test [27]. Assay wells contained 0.25% DMSO or less. 0.5 µCi of [^3^H]-hypoxanthine (19.4 Ci/mmol, GE Healthcare) was added after 48 hrs of contact, while parasite viability was determined by measuring [^3^H]-hypoxanthine incorporation after 18 hrs. Lysed parasites were harvested on filters using a Filtermate Harvester (Perkin Elmer) filled with 30 µl of scintillation liquid (Microscint, Perkin-Elmer), sealed, and counted in a TopCount NT Counter (Perkin Elmer).

### Drug inhibition of in vitro cultured *P. falciparum* growth

A two-step method was applied to synchronize the parasites. First, we used a magnetic cell sorter (VarioMacs, Miltenyi Biotec) that mainly retains schizonts [28], and second we used 5% sorbitol treatment 6 to 8 hrs later to selectively lyse mature stages [18]. Stage-dependent susceptibility was studied by adding MeSter at various concentrations when the parasites were in the ring (4 hrs after sorbitol treatment), trophozoite (20 hrs after sorbitol treatment), or schizont (33 hrs after sorbitol treatment) stages. Assays were initiated at 1% hematocrit and 0.6% parasitemia and MeSter was added for 6 hrs. Cells were then washed twice and resuspended in fresh complete medium. The course of *in vitro P. falciparum* growth inhibition was evaluated for these three stages. MeSter was added at 870 µM to synchronized cultures. After various lengths of incubation, cells were washed twice and resuspended in fresh complete medium. For both experiments, [^3^H]-hypoxanthine (0.5 µCi/well) was added at 52 hrs to monitor parasite viability. Reactions were stopped at 70 hrs and viability was evaluated for each stage and expressed as a percentage of the control (without drug).

### Effect of MeSter on *P. falciparum* morphology

*P. falciparum* parasites were synchronized with 5% sorbitol and MeSter was added 6 hrs later. Parasite morphology was evaluated by examination of blood smears every 6 hrs until reinvasion, i.e. 52 hrs post synchronization. Giemsa-stained blood smears were examined using a Nikon Eclipse 80*i* microscope and a Nikon DS-2Mv digital camera.

### Data analysis

Determination of enzyme kinetics, and IC_50_ and inhibition curves with MeSter and its analogues were generated using the Prism 5.0 software (GraphPad Inc.).

## Results

### Identification of a putative plasmodial stearoyl-CoA desaturase

**Figure 1:**
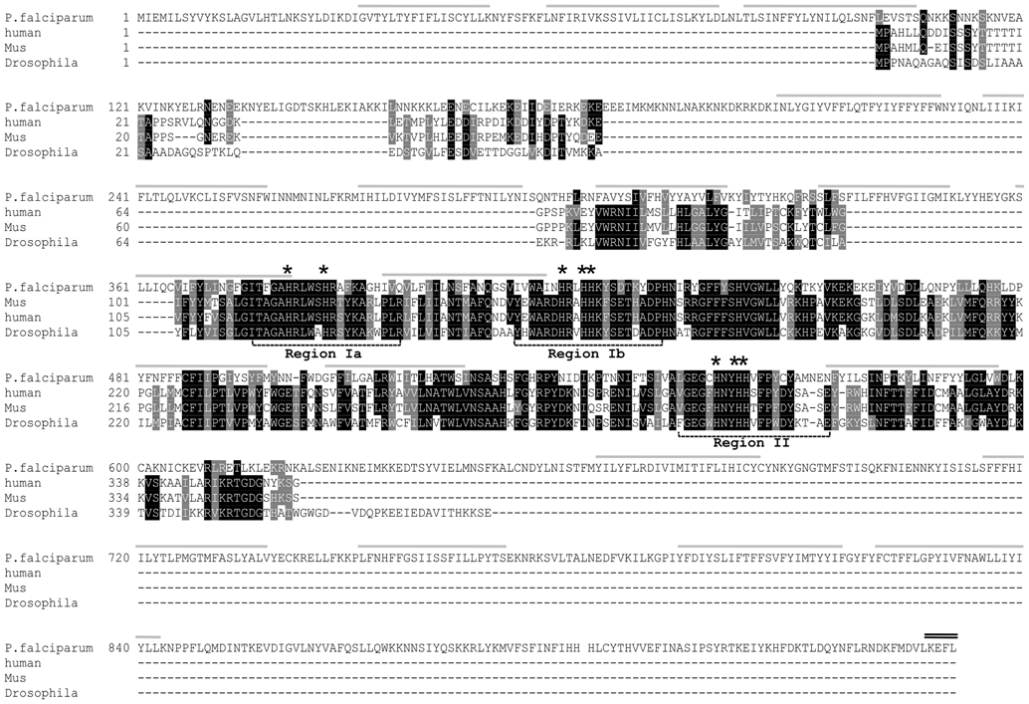
Aligned sequences of stearoyl-CoA Δ9-desaturases. Multiple sequence alignment was performed using CLUSTALW (http://www.ebi.ac.uk/Tools/clustalw2/index.html) and the BoxShade package (http://www.ch.embnet.org/software/BOX_form.html). *P. falciparum* SCD (GenBank accession number Q8I0W9) was compared with human SCD1 (accession number: O60427), *Mus musculus* SCD1 (accession number Q547C4) and *Drosophila melanogaster* SCD1 sequence (accession number Q7K4Y0). PfSCD transmembrane domains were predicted using the TMHMM package (http://www.cbs.dtu.dk/services/TMHMM-2.0/) and are shown in grey. Stars indicate the eight conserved His residues (2 in region Ia, 3 in region Ib, and 3 in region II). Identical amino acid residues are in black and homologous residues are shaded in grey. Gaps were introduced to facilitate the sequence alignment. The ER retention KDEL motif is indicated by a double black line.

*In silico* analysis of the *P. falciparum* 3D7 genome sequence [29] revealed the presence of a gene (*PFE0555w*) encoding a putative stearoyl-CoA Δ9-desaturase (PfSCD). This 2-exon gene encodes a protein of 949 amino acids (114 kDa, predicted pI of 9.2). Alignments of PfSCD with eukaryotic SCDs from human, *M. musculus* and *D. melanogaster* indicate that the malarial sequence is well conserved (Fig. 1). The identity rates between the central domain (residues 366 to 622) of PfSCD and these three eukaryotic orthologs are 41%, 38% and 42%, respectively. This central domain contains the catalytic domain of the SCD [13], comprising the three Histidine-Boxes designated region Ia, Ib and II (Fig. 1). All eight conserved His residues are essential for stearoyl-CoA desaturase activity, as mutations of any of these residues abrogate enzymatic activity [13]. Δ9-desaturase activity requires two co-acting enzymes, the cytochrome *b5* and NADPH cytochrome *b5*-reductase [30] that were also found to be present in the *P. falciparum* genome (accession numbers Q8I599 and Q8ID33, respectively). The cytochrome *b5* reductase provides cytochrome *b5* with electrons and protons extracted from NADPH. These attach to HisBox of SCD through two iron atoms and attaches two protons and two electrons to the fatty acyl-CoAs [31].

The plasmodial SCD appears as an unusually large SCD protein with the presence of additional N-terminal and C-terminal sequences (Fig. 1). These extra domains are also shared by other *Plasmodium* species. The overall amino acid sequence of PfSCD is well conserved in all plasmodial species with sequence identities of 74%, 71%, 71% and 70% compared to *P. vivax*, *P. yoelii*, *P. berghei* and *P. chabaudi* respectively (Figure S1). The ubiquitous presence of this protein in all sequenced *Plasmodium* species strongly suggests that it is probably fulfilling important physiological functions. An unexpected feature of PfSCD is the presence of eighteen predicted transmembrane domains (Fig. 1), in contrast with most eukaryotic SCDs that contain two to four transmembrane regions [32], [33], [34]. This high number of transmembrane domains may have hindered us from producing the full-length or the central domain of PfSCD in *E. coli*, despite repeated attempts.

### Δ9-desaturase expression and activity in *P. falciparum*

**Figure 2:**
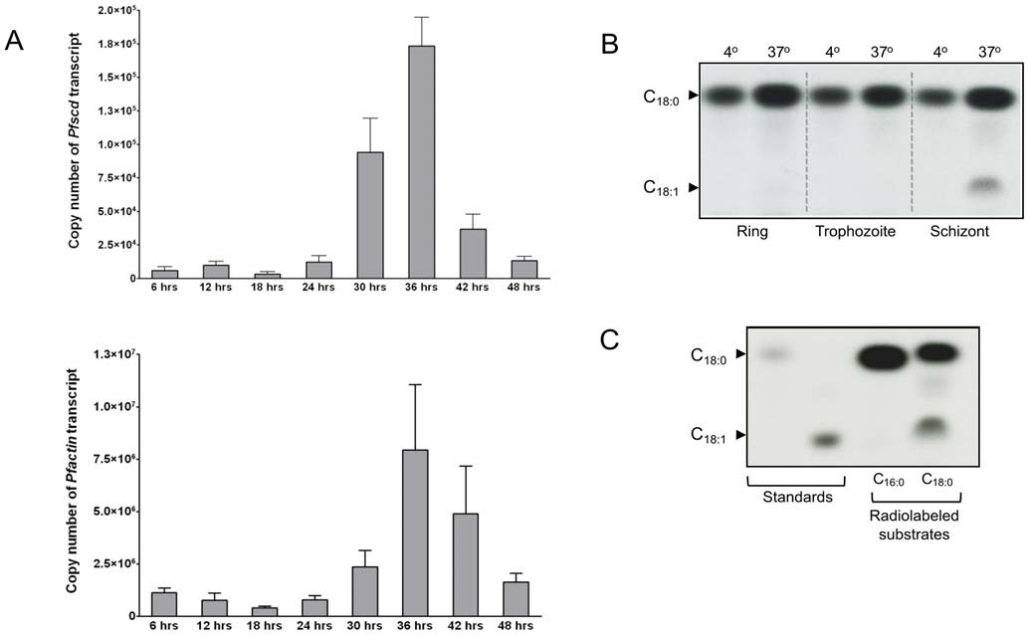
Stage-specific expression of *Pfscd* and stearoyl-CoA desaturase activity in *P. falciparum*. (A) Transcriptional profile of *Pfscd* (*PFE0555w*) as determined using quantitative RT-PCR. Total mRNA from synchronized *P. falciparum* was extracted every 6 hrs for one complete cycle (48 hrs). The mRNA was reverse-transcribed and the cDNA subjected to quantitative PCR using *Pfactin* as a reference gene. Results are expressed as transcript copy numbers, presented as the mean±SEM of 2 independent experiments performed in triplicate. (B) *In vivo* stearoyl-CoA desaturase activity. Various parasite stages were labeled with [^14^C]-stearic acid for 4 hrs either at 4°C or 37°C prior to extraction of the fatty acid methyl esters (FAMEs). Equal volumes of each sample (30%) were loaded on the TLC plate and resolved using petroleum ether/diethyl ether (17/3, v/v). C_18:0_ and C_18:1_ methyl esters were used as standards (indicated by arrowheads). Autoradiograms were exposed overnight to a Kodak Biomax MR film to reveal [^14^C]-labeled FAMEs. This autoradiogram is representative of results obtained in two independent experiments. (C) Substrate specificity of PfSCD. Schizonts were labeled with either radiolabeled palmitic acid (C_16:0_) or stearic acid (C_18:0_) for 4 hrs at 37°C. Following extraction, equal volumes were loaded on a TLC plate and FAMEs were resolved along with C_18:0_ and C_18:1_ methyl esters as standards using petroleum ether/diethyl ether (17/3, v/v). These were revealed after overnight exposure to a Kodak Biomax MR film.

Our first set of experiments confirmed that *P. falciparum* could synthesize oleic acid from exogenous stearic acid in non-synchronous IRBCs (data not shown). This finding agrees with Mi-Ichi *et al.* [35]. We next examined the *Pfscd* transcriptional profile through a complete erythrocytic cycle. Total mRNA was extracted from cultured, tightly synchronized *P. falciparum* 106/1 parasites every 6 hrs. Quantitative RT-PCR was performed on stage-specific mRNA and compared to a control *Pfactin* reference gene. As shown in Fig. 2A, *Pfscd* transcripts were predominantly expressed during the late trophozoite stage (between 30 and 42 hrs). There was a 53-fold increase of *Pfscd* transcript expression level between 18 and 36 hrs. In comparison, the *Pfactin* transcript expression level increased 19-fold between 18 and 36 hrs. Comparable results with respect to *Pfscd* mRNA induction were also obtained in cultured *P. falciparum* 3D7 parasites (data not shown). We subsequently investigated whether this could be correlated to a stage-specific Δ9-desaturase activity throughout the whole asexual parasitic cycle. For this experiment, different blood stages (rings, trophozoites and schizonts) were pulsed with [^14^C]-stearic acid. FAMEs were then extracted and analyzed by TLC on silver nitrate-impregnated plates to discern saturated from unsaturated FAs. Autoradiography presented clear evidence that no oleic acid was produced in IRBCs cultured at 4°C, whereas it was produced in schizonts at 37°C. No significant oleic acid levels were detected in rings and trophozoites (Fig. 2B). Schizonts produced 4.0 nmoles of oleic acid/10^10^ cells/hr, whereas rings produced 0.2 nmoles of oleic acid/10^10^ cells/hr. Overall, these results suggest that oleic acid is the product of an active developmental stage-specific process occurring at the late stage of the intraerythrocytic cycle.

Mammalian Δ9-desaturases have been the focus of considerable research in recent years [36], [37], [38]. Typically, mammals contain several SCDs that usually prefer stearoyl-CoA and palmitoyl-CoA as substrates. However, enzyme-substrate affinities and enzyme activities can differ within the same organism [39], [40]. To determine the preferred substrate of *P. falciparum* Δ9-desaturase, schizonts were labeled with 18 µM of either [^14^C]-stearic acid (C_18:0_; 1 µCi/ml) or [^14^C]-palmitic acid (C_16:0_; 1 µCi/ml). Autoradiograms of the FAs produced evidence of an unsaturated product, corresponding to oleic acid methyl ester, only when [^14^C]-stearic acid was supplied as a substrate (Fig. 2C). No palmitoleic acid (C_16:1_) was detected when parasites were provided with the substrate [^14^C]-palmitic acid. This indicates that, in contrast to most characterized mammalian Δ9-desaturases, the plasmodial Δ9-desaturase has a strong affinity for stearic acid with no observable activity on palmitic acid.

### Oleic acid synthesis in transgenic parasites overexpressing PFE0555w

**Figure 3:**
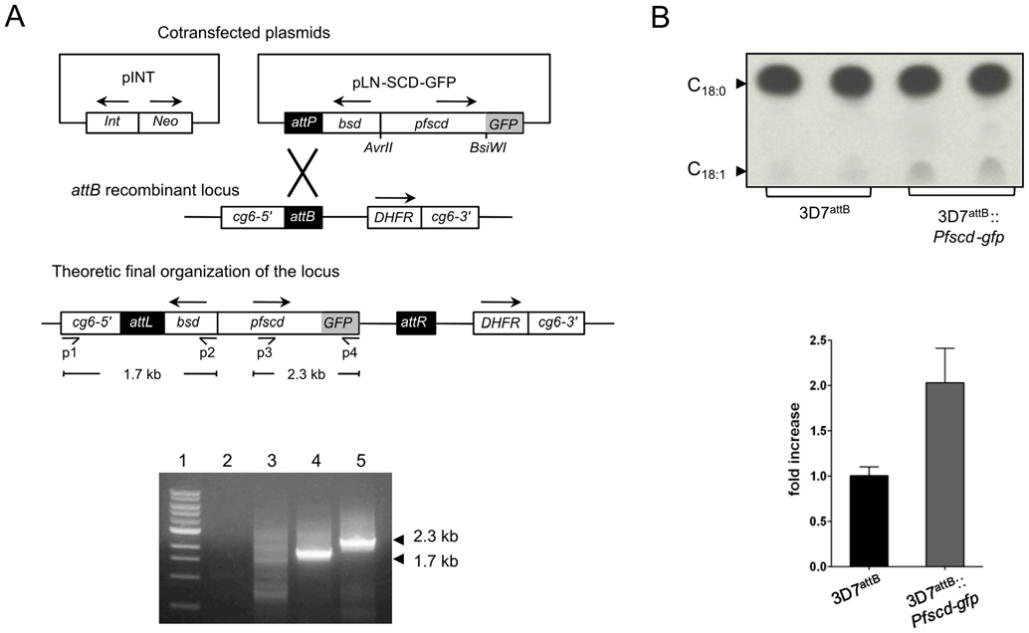
Stearic to oleic acid conversion in parasites overexpressing a *Pfscd-gfp* transgene. (A) Schematic diagram of the cotransfected plasmids, the recipient *cg6-attB* locus and the recombinant locus produced upon integration of the *Pfscd-gfp* cassette (upper panel). The lower panel represents integration of *Pfscd-gfp* into the *cg6-attB* locus of 3D7^attB^::*Pfscd*. Lane 1: 1 kb ladder. Lanes 2&4: integration of the blasticidin cassette into the locus was assayed by PCR using primers p1 and p2. Lanes 3&5: the presence of the complete *Pfscd-gfp* fusion was detected using primers p3 and p4. Lane 2&3: control 3D7^attB^ strain. Lane 4&5: 3D7^attB^::*Pfscd-gfp* line. (B) 3D7^attB^ and 3D7^attB^::*Pfscd-gfp* rings were labeled with 1 µCi of [^14^C]-stearic acid for 6 hrs at 37°C. Following extraction, equal counts (40,000 cpm) were loaded on a 10% silver nitrate-impregnated TLC plate. FAMEs were resolved using petroleum ether/diethyl ether (17/3, v/v) and revealed after overnight exposure to a Kodak Biomax MR film. The upper panel shows the fatty acid profile of two independent samples of each strain. Oleic acid methyl esters were scraped from the TLC plate and counted. Results are expressed as a fold increase of oleic acid production (normalized to a value of one in the 3D7^attB^ strain). The data in the lower panel represent the means of 4 experiments with standard deviations.

A transgenic *P. falciparum* cell line constitutively overexpressing a PfSCD-GFP fusion was generated using the site-specific integration system developed by Nkrumah *et al*. [17]. Both pINT (containing the integrase) and pLN-SCD-GFP (containing the *Pfscd-gfp* sequence along with the *attP* site) were co-transfected into the recipient 3D7^attB^ strain carrying the *cg6-attB* locus and selected using BSD and G418. This resulted in a transgenic line termed 3D7^attB^::PfSCD-GFP, in which the *Pfscd-gfp* sequence was integrated into the chromosomal *attB* site (Fig. 3A). Proper integration of the *Pfscd-gfp* transgene was confirmed by PCR using specific primers (Fig. 3A).

We first performed quantitative RT-PCR to compare the expression level of *Pfscd* transcripts in the 3D7^attB^::PfSCD-GFP and 3D7^attB^ strains. Parasites were synchronized, and total mRNA was extracted from early trophozoites (around 27 hrs). Quantitative RT-PCR indicated a 17% increase of *Pfscd* transcripts in the transgenic parasites containing the *Pfscd-gfp* construct, a value considered to be significant as revealed by the Student *t* test (P<0.01, data not shown). Second, both the parental and transgenic *P. falciparum* parasites were labeled with 18 µM [^14^C]-stearic acid at a late ring stage. Labeled FAs were extracted and analyzed by TLC as mentioned above. Rings were chosen due to their low oleic acid background level. As shown in Fig. 3B (upper panel), the overexpression of *PFE0555w* was associated with a significant increase in oleic acid production compared to the parental strain. Quantification of the radioactive spots corresponding to oleic acid methyl esters indicated that 3D7^attB^::*Pfscd-gfp* reproducibly exhibited a 2-fold increase in oleic acid synthesis with respect to 3D7^attB^ (Fig. 3B, lower panel). Similar results were observed following episomal expression of the transgene in the parasites (data not shown). Together, these results strongly suggest that *PFE0555w* encodes the plasmodial stearoyl-CoA desaturase.

### Kinetics of *P. falciparum* stearoyl-CoA Δ9-desaturase

**Figure 4:**
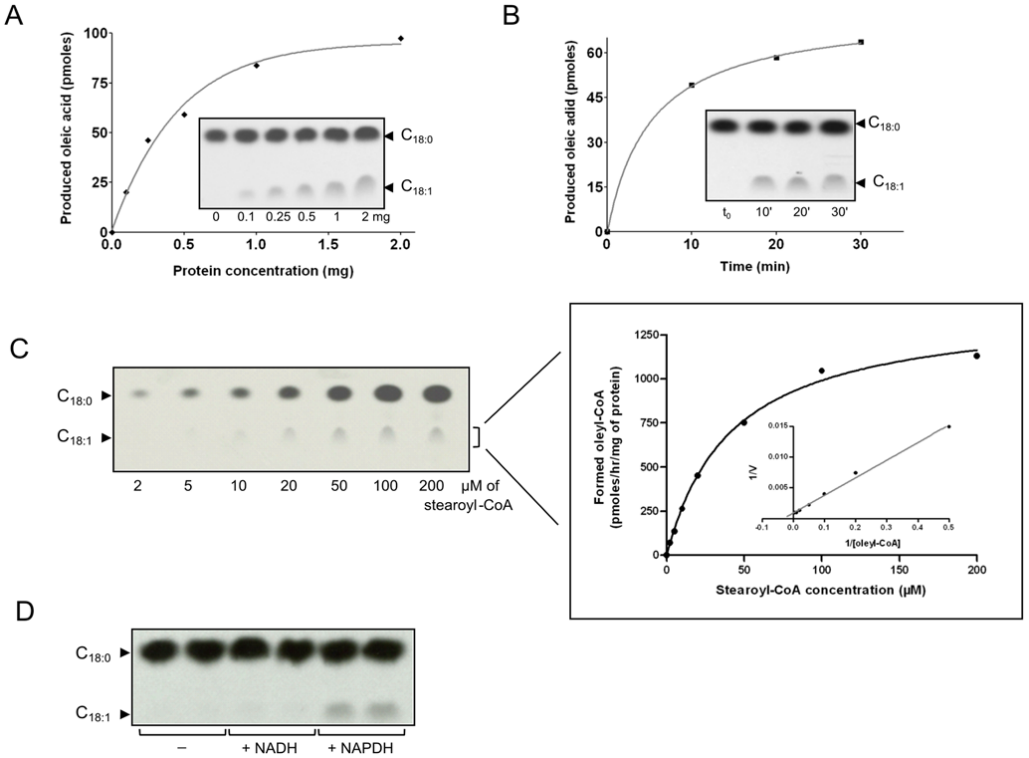
Stearoyl-CoA desaturase activity in a *P. falciparum* cell-free assay. (A) *In vitro* conversion of stearoyl-CoA to oleoyl-CoA in *P. falciparum* 3D7 schizont lysates, measured as a function of protein concentration. The reaction mix contained 0 to 2 mg of total lysate proteins, [^14^C]-stearoyl-CoA, and 2 mM NADPH, and was incubated for 30 min at 37°C. Following extraction of FAMEs, equal volumes of each sample (30%) were loaded on TLC plates and resolved using petroleum ether/diethyl ether (17/3, v/v). Radiolabeled FAMEs were revealed after overnight exposure to a Kodak Biomax MR film. Spots corresponding to oleic acid were scraped from the TLC plate and counted. Results are expressed as picomoles of oleic acid produced. (B) *In vitro* conversion of stearoyl-CoA as a function of reaction time. The reaction mix containing 500 µg total proteins, [^14^C]-stearoyl-CoA, and 2 mM NADPH, and was incubated at 37°C for various durations. Following extraction of FAMEs, equal volumes of each sample (25%) were loaded on TLC plates, resolved and revealed as described above. Results are expressed as picomoles of oleic acid produced. (C) *In vitro* conversion of stearoyl-CoA as a function of the substrate concentration. Increasing concentrations of [^14^C]-stearoyl-CoA, ranging from 2 to 200 µM, were mixed together with 500 µg total protein and 2 mM NADPH, and the reactions incubated for 5 min at 37°C. Following extraction of FAMEs, equal volumes of each sample (20%) were loaded on a TLC plate, resolved and revealed as described above. Oleic acid methyl esters were scraped from the TLC plate and counted to allow direct quantification of the enzymatic reaction. The specific activity is expressed in picomoles of oleoyl-CoA formed/hr/mg of input protein. The inset corresponds to the Lineweaver-Burke plot of the results. Results are expressed as a mean of 2 independent experiments. (D) NADPH dependency of the Stearoyl-CoA desaturase activity. Schizont membranes were incubated with [^14^C]-stearoyl-CoA either in the absence (−) or presence of 2 mM NADH or NADPH. Samples were processed as described in (A) and FAMEs were analyzed by TLC/autroradiography. Each condition was performed in duplicate.

To determine the desaturase kinetic parameters, we developed a cell-free assay using lysates of *P. falciparum* 3D7 schizonts incubated with NADPH and [^14^C]-stearoyl-CoA. After completion of the reaction, the mixture was hydrolyzed. From this, total FAs were extracted, and methyl-esterified prior to analysis by silver nitrate TLC. In a first set of experiments, we analyzed oleic acid production as a function of protein concentration (Fig. 4A). The curve appeared linear in a 0–500 µg schizont protein concentration range. Because 500 µg caps the linear phase of the curve, this amount was used in all subsequent experiments. The velocity of the reaction was determined by analyzing stearoyl-CoA conversion into oleoyl-CoA over time (Fig. 4B). Oleic acid was produced after 10 min, demonstrating very rapid reaction kinetics. Subsequent experiments concluded that the kinetic curve was linear during the first 5 min of the reaction (data not shown).

The kinetic parameters of stearoyl-CoA Δ9-desaturase activity were measured by incubating crude schizont extracts with increasing concentrations of radiolabeled stearoyl-CoA (Fig. 4C, left panel). The curve obtained from quantification of synthesized oleic acid displayed a typical shape, indicative of Michaelis-Menten kinetic characteristics. An apparent K_m_ value of 56.6 µM and a V_max_ of 1.15 µmol/hr/mg were determined as a mean of 2 independent experiments, with a variability of less than 25% between experiments (Fig. 4C, right panel). We note that these kinetic parameters reflect the SCD activity in schizont extracts, although one cannot exclude the possibility of separate enzymes that may contribute to the catalytic activity described.

We next investigated the NADPH and NADH dependency of this reaction. As shown in Fig. 4D, no oleic acid was produced in the absence of NADPH in the assay. When NADPH was substituted by NADH, only a very weak signal corresponding to oleic acid could be observed, suggesting that PfSCD is mainly dependent on NADPH. Because this assay was performed using parasite membranes, the presence of a Δ9-desaturase activity suggests that PfSCD is very likely to be associated to membranes. This prompted us to further investigate the localization of PfSCD in the parasite.

### Subcellular localization of PfSCD in *P. falciparum*

**Figure 5:**
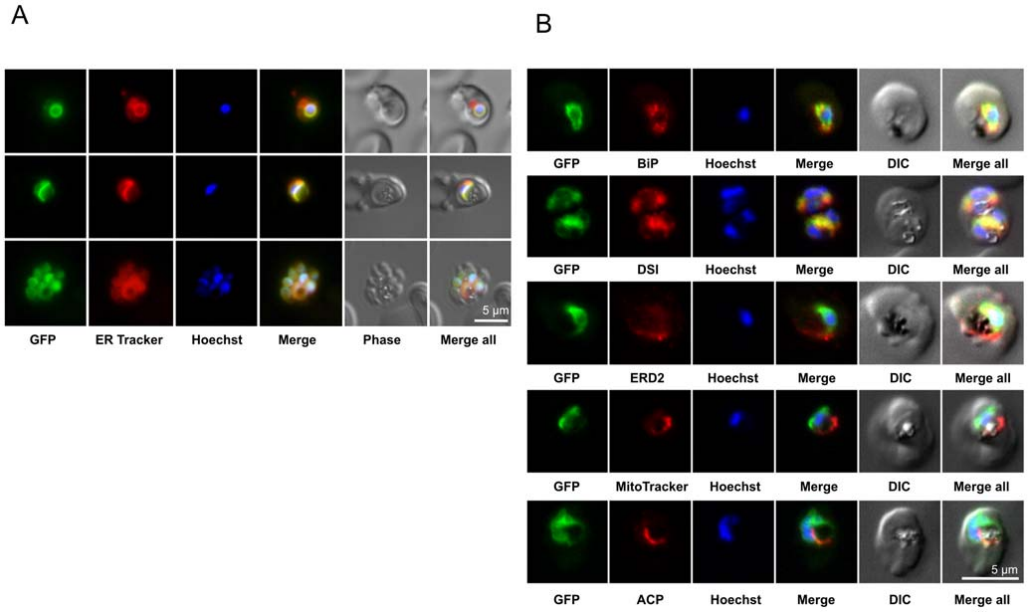
Localization of PfSCD-GFP in the parasite endoplasmic reticulum. (A) Direct fluorescence assays of PfSCD-GFP-expressing *P. falciparum* parasites (strain Dd2^attB^::*Pfscd-gfp*) with GFP (green), ER Tracker (red), and nuclear staining with Hoechst 33342 (blue). Images represent various stages of the erythrocytic cycle, respectively from top to bottom: early trophozoite, late trophozoite and segmented schizont. (B) Colocalization studies. PfSCD-GFP was colocalized with different compartment markers: ER (PfBip, PfDSI), *cis*-Golgi (PfERD2), mitochondria (MitoTracker Red), and apicoplast (PfACP).

The subcellular localization of PfSCD during intraerythrocytic infection was studied using PfSCD-GFP parasites. In most parasites, the green fluorescence surrounded the nucleus of the parasite stained in blue with Hoechst H33342 (Fig. 5A, GFP in green). This distribution is typical of the nuclear envelope and is considered as the main ER compartment in *Plasmodium* [41], [42], [43]. This was confirmed by the colocalization of the GFP green labeling with ER tracker red, a Glibenclamide-bodipy compound that shows a high affinity for the ER membrane (Fig. 5A). ER tracker has been widely used as an ER marker for fluorescence microscopy in various cell types. However, because ER tracker may also lead to non ER-membrane labeling depending on the cell type, we further investigated the sub-cellular localization of PfSCD by performing a detailed immunofluorescence analysis. As shown in Fig. 5B, PfSCD-GFP colocalized mostly with PfBiP and PfDSI (disulfide isomerase), two soluble ER markers [41], [43], therefore confirming the ER localization of PfSCD-GFP. In contrast, the GFP-tagged PfSCD protein did not colocalize with ERD2, a known marker of the *cis*-Golgi [41], [44]. The perinuclear labeling was consistent all throughout the intraerythocytic stage. Proliferation and branching were also observed during parasite maturation, in agreement with the description of the evolution of the ER during the parasite blood stage cycle [42], [45]. The GFP labeling was sometimes observed as a bright sub-domain of the nuclear envelope (Fig. 5A), or was closely apposed to the plasma membrane of the parasite, suggesting a dual localization to another organelle such as the mitochondria or the apicoplast, in addition to the ER. This issue was addressed by colocalization studies with MitoTracker red and apicoplast-specific anti-ACP antibodies. As shown in Fig. 5B, the mitochondria labeling appeared clearly distinct from the GFP signal at any erythrocytic stage. Moreover, the apicoplast did not colocalize with PfSCD-GFP, although it was often observed to be tightly apposed to particular domains of the GFP-labeled membrane. Taken together, these results confirm the ER membrane localization of PfSCD, which is consistent with the ER localization of SCDs previously described in mammals, such as mice and rats [34]. This is also consistent with the presence of a KEFL motif in the extreme C terminus of the protein (Fig. 1), which is closely related to the typical ER retention signal KDEL [46], [47].

### Inhibition of the Δ9-desaturase by methyl sterculate

**Figure 6:**
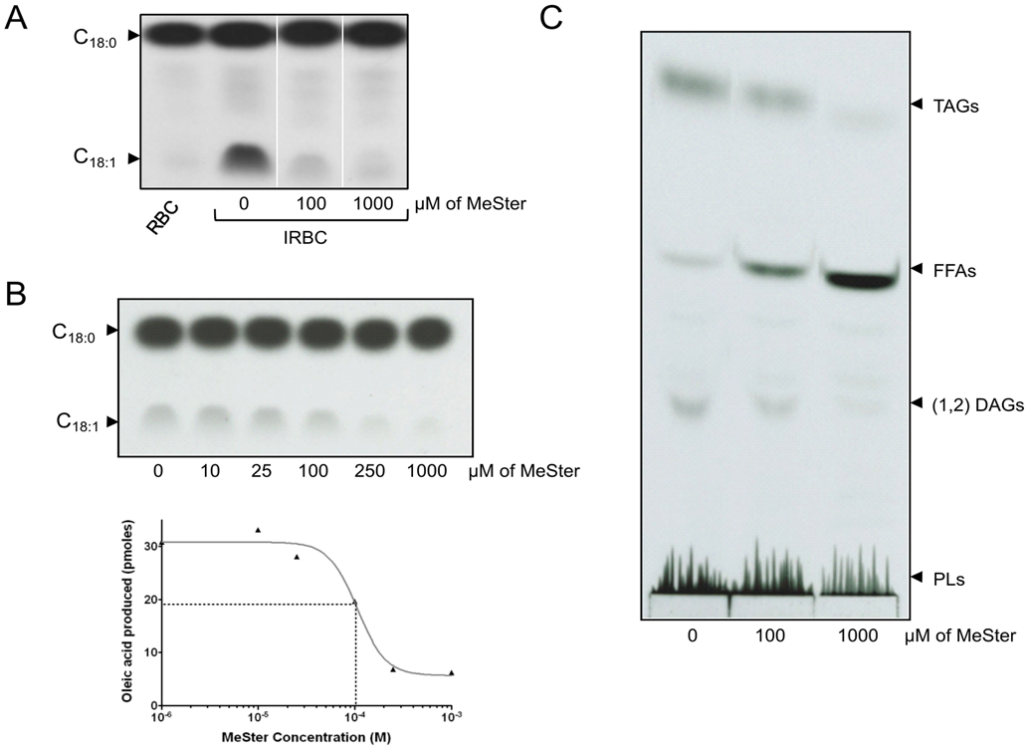
Inhibition of oleic acid synthesis by methyl sterculate. (A) *P. falciparum* 3D7-infected RBC were either left untreated or were treated for 1 hr with MeSter concentrations corresponding to 1× or 10× the IC_50_ value, and subsequently labeled with [^14^C]-stearic acid for an additional 4 hrs. Non-infected red blood cells (RBC) were used as a negative control. Following extraction, FAs were methyl-esterified and equal volumes were loaded on a TLC plate and resolved as described in the Experimental Procedures. (B) Inhibition of the stearoyl-CoA desaturase activity in parasite lysates. Crude extracts (500 µg total protein) were preincubated for 10 min at 37°C with 2 mM NADPH, in the presence of increasing concentrations of MeSter as indicated, prior to the addition of [^14^C]-stearoyl-CoA. After an additional 5 min incubation at 37°C, FAMEs were extracted, methyl-esterified and equal amounts of radioactivity counts were loaded on a TLC plate. The upper panel corresponds to the autoradiogram and the lower panel represents the curve obtained after scrapping the OAME spots, illustrating the effect of MeSter on oleic acid production. (C) Effect of MeSter treatment on parasite lipid composition. *P. falciparum* IRBCs were treated or untreated with MeSter, corresponding to 1× or 10× the IC_50_ value, for 1 hr. Parasites were subsequently labeled with [^14^C]-stearic acid for an additional 4 hrs. Following extraction, the same amounts of radioactivity were loaded and neutral lipids were resolved as described in the Experimental Procedures. DAGs, diacylglyerols; FFA, free fatty acids; PLs, phospholipids; TAG, triacylglycerols.

To evaluate the essentiality of oleic acid during intraerythrocytic growth, we tested selective Δ9-desaturase inhibitors. Sterculic acid is a cyclopropene-containing FA, naturally found in plants such as *Sterculia foetida*, which has been shown to specifically inhibit Δ9-desaturation of stearic to oleic acid [48], [49]. Recent work has revealed potent antimycobacterial activity of sterculic acid targeting the stearoyl-CoA desaturase DesA3 in *Mycobacterium tuberculosis* [15]. Herein, we examined whether its methyl ester (methyl sterculate, MeSter) might inhibit oleic acid biosynthesis by targeting the *P. falciparum* stearoyl-CoA desaturase. In the first set of experiments, schizonts were treated with increasing concentrations of MeSter and labeled with [^14^C]-stearic acid. FAMEs were then extracted and developed by silver-nitrate TLC. As expected, IRBCs produced large amounts of radiolabeled oleic acid in the absence of MeSter (Fig. 6A). This signal was strongly reduced when schizonts were treated with 100 µM MeSter, and was virtually abrogated in the presence of 1 mM MeSter. No oleic acid production was observed in uninfected RBC. These results suggest that MeSter inhibits oleic acid synthesis *in vivo*.

We subsequently employed a cell-free assay to determine whether MeSter directly targets PfSCD. In this assay, schizont lysates were incubated with [^14^C]-stearoyl-CoA in the presence of increasing concentrations of MeSter. FAMEs were then extracted and developed by silver-nitrate TLC. Fig. 6B demonstrates that PfSCD was inhibited by MeSter in a dose-dependent manner. Quantitative analysis of the radiolabeled spots corresponding to oleic acid enabled us to derive an IC_50_ value for MeSter of 105 µM (Fig. 6B).

In further studies, we investigated whether newly synthesized oleic acid could be incorporated into more complex parasite lipids such as neutral lipids or phospholipids. With MeSter specifically inhibiting the conversion of stearic acid to oleic acid, we reasoned that a comparative analysis of the total lipid profile of untreated and MeSter-treated parasites would identify the lipids that contain stearoyl-originating oleic acid. Schizonts were labeled with [^14^C]-stearic acid, treated or untreated with MeSter, and the total lipids were extracted and resolved by TLC using heptane/diisopropyl oxide/acetic acid. Fig. 6C indicates that high doses of MeSter correlated to low signals corresponding to triacylglycerol (TAG), 1,2-diacylglycerol (DAG) and phospholipids (PL), when compared to untreated control cells. Equal counts were loaded in each lane for direct comparison of the lipid profiles, indicating that most of the counts were recovered in the free FAs in parasites treated with high concentrations of MeSter. Together, these results suggest that newly synthesized oleic acid is incorporated into TAGs, 1,2-DAGs and phospholipids. Additional experiments revealed that newly synthesized oleic acid was present in phosphatidylcholine, phosphatidylethanolamine, phosphatidylinositol and to a lesser extent phosphatidic acid (data not shown). These data provide evidence that oleate represents a major FA in plasmodial phospholipids and is actively incorporated into parasite lipids [50], [51], [52].

### Antiplasmodial activity of methyl-sterculate

**Figure 7:**
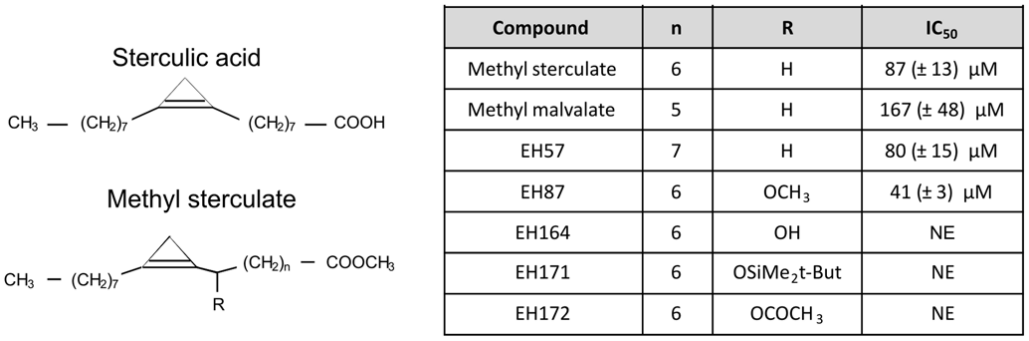
Structure and antimalarial activity of methyl sterculate and its analogs. Chemical structure of sterculic acid, as well as methyl sterculate (MeSter) and some derivatives. Inhibition of the intraerythrocytic growth of *P. falciparum* 3D7 by each compound is expressed as the mean±SEM IC_50_ value (in µM). Each compound was tested in duplicate on three separate occasions. NE, no effect on parasite viability was observed at the highest concentration tested (1 mM).

The mechanism of action of MeSter prompted us to investigate whether this inhibitor was active against *P. falciparum* asexual blood stage parasites. The structures of sterculic acid, the parental natural compound and its methyl ester (MeSter) and related analogues are depicted in Fig. 7. Our initial work using various concentrations of human serum in the medium (ranging from 2% to 20%) indicated that the maximal antiplasmodial activity of MeSter was obtained in the presence of 4% serum (data not shown), corresponding to approximately 0.3 µM oleic acid in the culture medium. Therefore, the 4% serum concentration was used in all subsequent experiments. Using a modified Desjardins test [27], MeSter was found to exhibit significant antimalarial activity against 3D7 parasites, with an IC_50_ value of 87 µM. The antimalarial activity of MeSter analogs is presented in Fig. 7. Among all the analogs, the methoxy-containing analogue EH87 was found to be the most active, with an IC_50_ value of 41 µM.

To link the antimalarial activity of MeSter to its capacity to inhibit Δ9-desaturase-dependent oleic acid biosynthesis, we generated an isobologram to test for interactions between the inhibitor (MeSter) and the final reaction product (oleic acid) [53]. The graph presented in Figure S2 (left panel) is strongly suggestive of antagonism between the two molecules [54]. This observation is also supported by the fact that supplementation with exogenous oleic acid (25 µM) partially reverses the antimalarial activity of MeSter (Figure S2, right panel). The IC_50_ value of oleic acid against *P. falciparum* was found to be around 150 µM (data not shown), consistently with a previous report [55]. Because the fatty acid composition is extremely important to provide the appropriate physical and biological membrane properties, high concentrations of oleic acid undoubtedly alter the fatty acid composition equilibrium, ultimately leading to parasite death.

**Figure 8:**
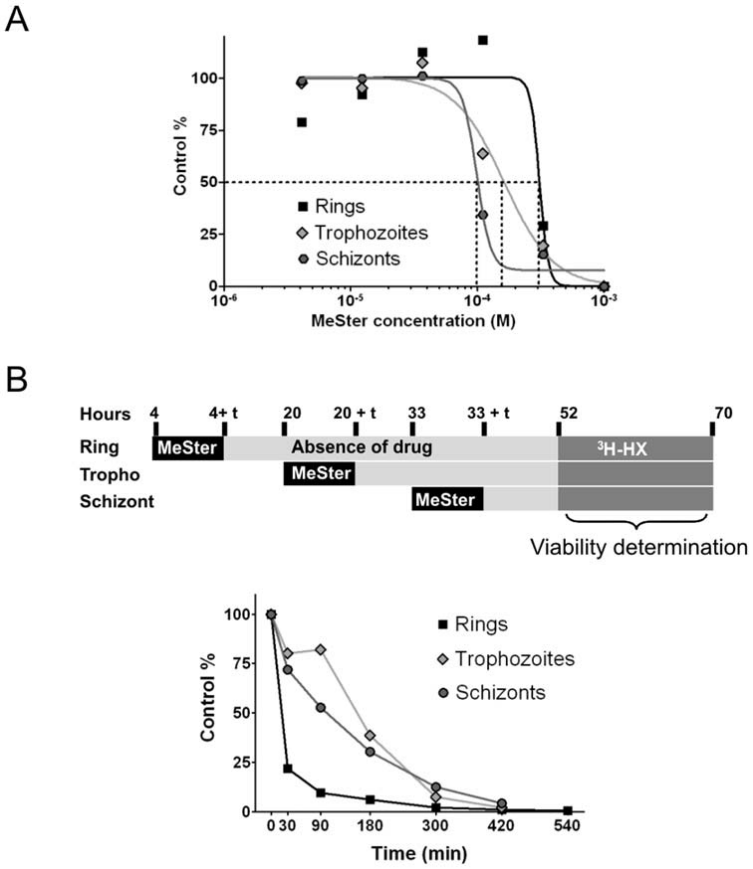
Life cycle-dependent sensitivities of *P. falciparum* to methyl sterculate throughout asexual blood stage growth. (A) Stage-specific MeSter IC_50_ value. This value was determined using a range of MeSter concentrations at each stage, following 6 hrs of parasite exposure to the drug. The medium was then removed, cells were washed twice, and fresh medium was added. Viability was determined after 52 hrs by measuring the parasite ability to incorporate [^3^H]-hypoxanthine for a further 18 hrs. Results are expressed as the mean of 2 independent experiments. (B) Time-course of parasite growth inhibition by MeSter. The upper panel is a schematic representation of the experimental protocol. Drug was added at each developmental stage for the indicated times. The medium was then removed, cells were washed twice, and fresh medium was added. Viability was determined after 52 hrs by measuring the ability of the parasites to incorporate [^3^H]-hypoxanthine for a further 18 hrs. The graph in the lower panel illustrates the sensitivity of each parasite stage to MeSter as a function of the duration of exposure to compound. Results are expressed as the percent [^3^H]-hypoxanthine incorporation compared to non-treated parasites, and reflect the mean of 2 independent experiments.

In order to examine the stage-specific sensitivity of *P. falciparum* to MeSter, parasite cultures were synchronized and the effect of the inhibitor was tested throughout the cycle. In this assay, various concentrations of MeSter were delivered to parasites for a fixed period of 6 hrs. Cells were then washed and resuspended in fresh medium, and the viability determined at 52 hrs. The resulting curves are depicted in Fig. 8A, and produced IC_50_ values of 310 µM, 162 µM and 99 µM for rings, trophozoites and schizonts, respectively. These results indicated that all stages were sensitive to killing by MeSter.

The potential of MeSter to inhibit intraerythrocytic growth of the parasite was further evaluated in a time-course experiment. This was achieved following a protocol illustrated in Fig. 8B (upper panel). Briefly, we performed a time-course analysis of drug contact on each parasite stage by delivering the drug to parasites at 10X IC_50_ for various times (ranging from 30 min to 9 hrs). After this time of contact, the drug was washed out, parasites were resuspended in fresh medium, and the parasite viability checked after the end of the first complete cycle at time 52 hrs. The graph in Fig. 8B shows the viability curves obtained for each stage. All curves reached the X-axis of the graph or 0% control after 7 hrs of contact, indicating that MeSter at 10X IC_50_ irreversibly affected parasite viability, regardless of the stage. Thus, MeSter produced a parasitocidal effect against cultured *P. falciparum* after 7 hrs. The fastest cidal effect was observed with ring stage parasites (viability was decreased by 80% after 30 min of contact with the drug), indicating that the drug impacted these early stages more rapidly than the mature stages.

### Morphological evolution of MeSter-treated parasites

**Figure 9:**
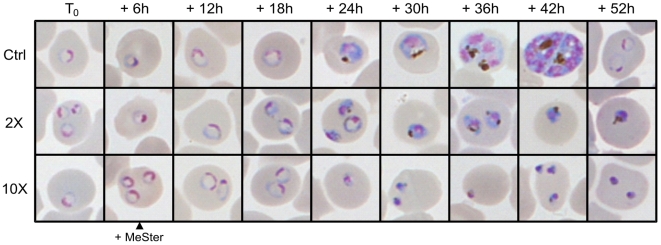
Effect of MeSter on parasite morphology. MeSter at 2-fold and 10-fold the IC_50_ was added to *P. falciparum* 6 hrs after synchronization (T_0_). Giemsa-stained blood smears were performed every 6 hrs until reinvasion of the control dish 52 hrs later. Representative images are shown.

Morphological and kinetic changes induced by treatment of the IRBCs with MeSter were monitored by Giemsa-staining of blood smears. MeSter was added 6 hrs after synchronization at 2-fold and 10-fold the IC_50_ value. Representative images of the morphological evolution are presented in Fig. 9. In control cells, ring stage parasites (T_0_–18 hrs) show a large, hemoglobin-containing vacuole with no pigment visible in light microscopy. Between 18 and 24 hrs post-invasion, the pigment appears and the size of the food vacuole is reduced. From 36 hrs post-invasion, they undergo schizogonia, the nucleus starts to divide and the pigment increases in size. No significant changes were observed in treated parasites during the first 18 hrs, i.e. 12 hrs after drug addition. However, important abnormalities began to appear after 24 hrs. At a low dose of MeSter (2X IC_50_), the appearance of the pigment and the reduction of the hemoglobin-containing vacuole are delayed, suggesting a slowdown of growth during the ring stage. From 30 up to 52 hrs post-invasion, treated parasites remain morphologically similar to untreated parasites at 30 hrs, suggesting that the development is blocked in the mid-trophozoïte stage. High doses (10X IC_50_) of MeSter led to pycnotic parasites, suggesting that they had already engaged a cell death process.

## Discussion

Substantial evidence indicates that phospholipid and neutral lipid biosynthesis in *P. falciparum* asexual blood stages relies primarily on the uptake of FAs from host plasma [2], [4], [12], [50]. Biochemical studies with *Plasmodium*-infected erythrocytes have demonstrated that intracellular transacylation reactions or release of FA moieties from intact phospholipid molecules do not occur to a significant level [56]. Our study extends these observations by finding that *P. falciparum* is able to unsaturate scavenged stearic acid, producing oleic acid, and that the Δ9-desaturase activity appears essential for parasite blood stage growth.

Here, we have reported and characterized the activity of *P. falciparum* stearoyl-CoA Δ9-desaturase (PfSCD), which catalyzes the conversion of stearoyl-CoA to oleoyl-CoA. Our genome mining studies identified PFE0555w as a putative SCD. This protein showed an unusually high number of predicted transmembrane domains when compared to most mammalian SCDs. We posit that this was, at least in part, responsible for the failure to express this protein in *E. coli*. Several lines of evidence nevertheless indicate that this protein represents the appropriate candidate. First, PFE0555w exhibits strong similarity with other mammalian SCDs, although it possesses additional N-terminal and C-terminal extensions. Examination of the primary sequence reveals the presence of the three His boxes in regions Ia, Ib and II, a signature feature of stearoyl-CoA desaturases [13]. Second, transgenic parasites overexpressing a PFE0555w-GFP fusion localized this protein to the ER, a feature also shared with mice, rat and yeast SCDs [32], [34]. It is noteworthy that PFE0555w was originally predicted to be an apicoplast protein [57], however we find that the sequence lacks the typical apicoplast targeting signals. Third, we have cloned and overexpressed the central domain of PFE0555w (residues 262 to 647, termed PFE0555w-C) in *M. bovis* BCG, an organism whose stearoyl-CoA desaturase (DesA3) has been reported to be a target of the antitubercular thiourea drug isoxyl that inhibits oleic acid production [15]. We observed a two-fold decrease in susceptibility to isoxyl in *M. bovis* BCG strains overexpressing plasmodial PFE0555w-C, when compared to the control strain (Figure S3). These results are similar to those obtained by Phetsuksiri *et al*. [15], who reported that a *M. bovis* BCG strain overexpressing DesA3 exhibited a two-fold increase in resistance against isoxyl. This implies that the central domain of PFE0555w, like DesA3, represents a functional target of isoxyl in mycobacteria. These findings strongly suggest that PFE0555w shares a similar activity with the mycobacterial stearoyl-CoA desaturase DesA3 and lead us to posit that PFE0555w is PfSCD. Finally, functional analysis of transgenic parasites overexpressing *PFE0555w* clearly demonstrated a significant 2-fold increase in stearic acid to oleic acid conversion at the late ring stage, compared to the parental strain. It is noteworthy that SCDs are usually part of a multiprotein complex, along with cytochrome *b5* and NADPH-cytochrome *b5* reductase. Therefore, overexpression of only one partner of this complex, such as PFE0555w, may limit an important increase in SCD activity.

Previous reports have failed to find evidence that intraerythrocytic *P. falciparum* parasites can elongate or desaturate fatty acids [5], [12]. In this study, we have developed a sensitive *in vitro* assay that enabled us to observe the conversion of stearic acid to oleic acid in both *P. falciparum* crude extracts and membranes. Although mammalian SCDs desaturate several substrates such as palmitoyl-CoA and stearoyl-CoA [40], PfSCD appears to be highly specific to stearoyl-CoA, as no palmitoleoyl was formed using palmitate as a primer. Moreover, our study revealed that stearoyl-CoA desaturase activity was predominant at the later stages of the erythrocytic cycle, especially in schizonts, a stage characterized by an extensive build-up of membranes required for the formation of merozoites. This is in agreement with earlier work showing that parasite lipid biosynthetic activities are maximal at the schizont stage [50]. The increased SCD activity is also evidenced by the high induction of specific *Pfscd* transcripts in late trophozoites (harvested 36 hrs post-invasion), producing a 50-fold increase compared to the ring stage.

We provide here clear evidence that PfSCD is associated with the *P. falciparum* ER. This indicates that desaturation of stearoyl-CoA does not occur in the apicoplast, the site of FA elongation by the functional FAS-II system [8], [9]. However, it remains possible that FAs, such as oleic acid, may be produced using a system that is different from the conventional type I or type II fatty acid synthases, at least during the asexual erythrocytic cell cycle. Indeed, recent work indicates that although the FAS-II system is dispensable during the asexual blood stages, it is required for FA synthesis during the infectious liver stage [10]. This was demonstrated in both *P. falciparum* and *P. berghei* using disruption mutants of the *fabI* gene encoding the FAS-II enzyme enoyl ACP reductase. Evidence that the FAS-II pathway is not active in asexual blood stages, but is required for the maturation of liver stages, was also provided by studies of *P. yoelii* knockout parasites lacking the *fabB/F* or *fabZ* genes [11]. These results imply the existence of a separate mechanism of FA synthesis during asexual blood stage development. Interestingly, an elegant study recently reported that trypanosomes can synthesize FAs using a microsomal elongase pathway that differs from FAS-I or FAS-II [58]. Similar elongases are encoded by the *P. falciparum* genome [(*PfD1* (NP_703294), *PfD2* (NP_704739), and *PfE* (XP_966049)] [59], and their role in FA biogenesis remains to be investigated. It is tempting to speculate that the ER, rather than the apicoplast, represents the site of FA elongation and desaturation in *Plasmodium* parasites during intraerythrocytic growth. These FAs could then directly be utilized for the synthesis of phospholipids and membrane biogenesis. Interestingly, the *P. falciparum* PfGatp protein, which catalyzes the first step of phospholipid biosynthesis by controlling the acylation of glycerol 3-phosphate at the *sn*-1 position, is also an integral membrane protein resident in the ER [60].

Cyclopropene FAs, such as sterculic acid, are known to be specific inhibitors of Δ9-desaturases [48], [49]. Our experiments with methyl sterculate demonstrated a pronounced inhibition of Δ9-stearoyl-CoA desaturase activity in *P. falciparum*. These data indicate that MeSter not only inhibited the production of oleic acid in living parasites in a dose-dependent manner, but also inhibited Δ9-stearoyl-CoA desaturase activity in lysates with an IC_50_ value similar to that required for 50% inhibition of parasite growth. This prompted us to analyze the fate of newly formed oleic acid in parasite lipids. Results showed that oleic acid was incorporated into essentially all lipids, including neutral lipids and phospholipids.

Although the molecular mechanisms by which MeSter leads to parasite cell death remains obscure, we propose several hypotheses to explain the effect of oleic acid synthesis inhibition. The first is that MeSter considerably depletes parasites of newly synthesized oleic acid, thus changing the stearic/oleic acid ratio. This imbalance in the saturated/unsaturated lipid ratio could have important consequences regarding membrane fluidity and cellular trafficking within the parasite. Therefore, the antimalarial activity of MeSter would directly depend on a profound alteration of membrane fluidity. An alternative hypothesis is that while newly synthesized oleic acid is incorporated in neutral lipids and phospholipids, it is also required for another important lipid that is involved in a specific metabolic process or signal transduction event. Interestingly elucidation of the *P. falciparum* glycosylphosphatidylinositol (GPI) moieties that anchor parasite proteins to the surface of the asexual blood stage merozoite reveal these to be enriched in C_16:0_ (palmitic acid), C_18:0_ (stearic acid) and C_18:1_ (oleic acid), with each GPI moiety typically containing two saturated and one monounsaturated FA [61]. A change in the stoichiometry of these unsaturated and saturated FA through inhibition of PfSCD could therefore be rapidly detrimental to parasite growth.

At low concentration, MeSter induces a delay in the evolution of the ring form followed by an arrest of the parasite development around the mid-trophozoïte stage. This growth defect may be due to an alteration of hemoglobin digestion. The association of lipid bodies and substantial amounts of DAG and TAG with the food vacuole suggests that neutral lipids and their precursors may also assist in the crystallization of haematin, and play a role in heme detoxification during the early trophozoite stage [62]. Since newly synthesized oleic acid is incorporated into most lipids, including DAG and TAG (Fig. 6C), it can then be speculated that impairment of oleic acid synthesis may have consequences on the content or composition of the food vacuole lipids, thus affecting hemozoin formation. However, this requires to be further investigated.

Assays to monitor the antimalarial activity of MeSter indicated that all erythrocytic stages were sensitive to inhibition by this compound. Time-course experiments indicated that the ring stage is more rapidly inhibited, compared to the other stages. This is a particularly unexpected observation as relatively few antimalarial agents, notably artemisinin [63], triclosan [64], and the choline analog T3 [65], show rapid onset of inhibition of ring stage parasites. Most antimalarials, including chloroquine and the antifolates, are usually more effective during late erythrocytic stages [63], [66], [67]. However, the reason why rings are more rapidly inhibited than the other stages is presently not known.

We also designed and synthesized several MeSter analogues for structure-function studies. Methyl malvalate, which has cyclopropene at the 8,9- position in the chain relative to the ester, showed an IC_50_ value twice that of MeSter. In contrast EH57, which has the cyclopropene at the 10,11 position, had an IC_50_ value similar to MeSter. An earlier study of related compounds showed that desaturase activity required at least one of the carbons of the cyclopropene to be in the 9 or 10-position [68]. Among the analogues tested here, only EH87 (with a methoxy group in position 8 as a racemic mixture) reproducibly demonstrated a two-fold improved IC_50_ value over MeSter. Yet the closely related alcohol EH164, acetate EH172 and silyl ether EH171 showed no activity in our assay. With the latter compounds, this could reflect a steric effect, although the inactivity of EH164 remains to be accounted. These results raise the possibility of designing more potent sterculic acid mimics and in particular, studying the effect of the stereochemistry of substituents adjacent to the cyclopropene ring.

In conclusion, oleic acid synthesis in *P. falciparum* is strongly supported by our molecular and biochemical data. The inhibition data highlight the essential nature of this desaturation product and the potential of generating sterculic acid analogs with increased antimalarial activities. Given the requirements for new antimalarials with novel modes of action, we propose that fatty acid metabolism, which appears to achieve a stage-specific balance between salvage from the host and modification by the parasite, represents an attractive process with significant therapeutic value.

## Supporting Information

Figure S1 Multiple alignment of putative plasmodial SCDs.
(0.15 MB DOC)

Figure S2 Isobologram plots of MeSter plus oleic acid tested against P. falciparum cultures.
(0.07 MB DOC)

Figure S3 Increased resistance to isoxyl of a Mycobacterium bovis BCG strain overexpressing the central domain of P. falciparum PFE0555w.
(0.56 MB DOC)

Methods S1 Preparation of cyclopropenes.
(0.10 MB DOC)
