# Supplementary material for: Oleic Acid Biosynthesis in Plasmodium falciparum: Characterization of the Stearoyl-CoA Desaturase and Investigation as a Potential Therapeutic Target
Source: PLoS One. 2009 Sep 3;4(9):e6889. doi: 10.1371/journal.pone.0006889 (PMC2731242; doi:10.1371/journal.pone.0006889)
Supplement: Figure S1 — Multiple alignment of putative plasmodial SCDs. (0.15 MB DOC) [file pone.0006889.s001.doc]

**Figure S1. Multiple alignment of putative plasmodial SCDs.** These were constructed using CLUSTALW (<http://www.ebi.ac.uk/Tools/clustalw2/index.html>) and the BoxShade package (<http://www.ch.embnet.org/software/BOX_form.html>). *P. falciparum* SCD (accession number Q8I0W9), *P. vivax* (accession number EDL44116), *P. yoelii* (accession number EAA16836), *P. berghei* (accession number CAH96615) and *P. chabaudi* (accession number CAH77162) putative SCD sequences were acquired from GenBank. Identical amino acid residues are in black and homologous residues are shaded in grey. Gaps were introduced to facilitate the sequence alignment.
