# Supplementary material for: Oleic Acid Biosynthesis in Plasmodium falciparum: Characterization of the Stearoyl-CoA Desaturase and Investigation as a Potential Therapeutic Target
Source: PLoS One. 2009 Sep 3;4(9):e6889. doi: 10.1371/journal.pone.0006889 (PMC2731242; doi:10.1371/journal.pone.0006889)
Supplement: Figure S2 — Isobologram plots of MeSter plus oleic acid tested against P. falciparum cultures. (0.07 MB DOC) [file pone.0006889.s002.doc]

**figure S2. Isobologram plots of MeSter plus oleic acid tested against *P. falciparum* cultures.** MeSter and free fatty acids (FFA) were added to parasite cultures either alone or in combination at different ratios at time 0. After 48 hr, [^3^H]-hypoxanthine was added and cell viability was measured at 66 hr. Interactions between MeSter and FFA were determined according to Berenbaum [1]. All concentration points of each compound were determined as the fraction of the IC_50_ (FIC_50_) values determined when the compound was tested alone. The type of interaction was determined by calculating the sum of IC_50_ of the two compounds as described earlier [2]: ΣFIC_50_ = FIC_50_ MeSter + FIC_50_ FFA. Additivity of the drugs was deduced if the ΣFIC_50_ was between 0.5 and 2, synergism was deduced if ΣFIC_50_ was <0.5 and antagonism was deduced if ΣFIC_50_ was ≥2.

Each IC_50_ obtained with MeSter was expressed as a fraction of the control IC_50_ of MeSter (Fractional IC_50_ or FIC_50_), and each concentration of oleic acid was also expressed as a FIC_50_. The left panel shows one typical experimental result (full line) from the six independent experiments. The dotted black line reflects additivity and the dotted grey line reflects antagonism (ΣFIC_50_ ≥2). The mean sum of the IC_50_ values around 2 is indicative of a significant antagonistic effect between MeSter and oleic acid.

The right panel represents a MeSter susceptibility curve of *P. falciparum* 3D7 cultured in the absence (black curve) or presence (grey curve) of 25 μM exogenous oleic acid.
