# Supplementary material for: Oleic Acid Biosynthesis in Plasmodium falciparum: Characterization of the Stearoyl-CoA Desaturase and Investigation as a Potential Therapeutic Target
Source: PLoS One. 2009 Sep 3;4(9):e6889. doi: 10.1371/journal.pone.0006889 (PMC2731242; doi:10.1371/journal.pone.0006889)
Supplement: Figure S3 — Increased resistance to isoxyl of a Mycobacterium bovis BCG strain overexpressing the central domain of P. falciparum PFE0555w. (0.56 MB DOC) [file pone.0006889.s003.doc]

**figure S3. Increased resistance to isoxyl of a *Mycobacterium bovis* BCG strain overexpressing the central domain of *P. falciparum* PFE0555w.** The central domain of PFE0555w (PFE0555w-C) was PCR amplified from *P. falciparum* 3D7 cDNA using with the following primers: 5'-ccg gat cca tga ata taa att tat tca aaa gaa tga tac-3' (*Bam*HI site underlined) and 5'-ccc aag ctt gaa aga att cat taa ttc tat a-3' (*Hin*dIII site underlined). The PCR product was then restricted with *Bam*HI and *Hind*III and cloned in pVV16 digested with the same enzymes. This plasmid is an *E. coli*-mycobacterial shuttle vector that carries a kanamycin resistance cassette and that allows expression of genes placed under the control of the mycobacterial *hsp60* promoter [3]. The resulting plasmid pVV16 ::*PFE0555w-C* was introduced into *M. bovis* BCG Pasteur and recombinant clones were selected on Middlebrook 7H11 solid medium supplemented with ADC (albumin-Dextrose, Catalase) and 25 μg/ml kanamycin.

The susceptibilities of *M. bovis* BCG strains containing either pVV16 (control strain) or pVV16 ::*PFE0555w-C* were evaluated by streaking aliquots of mid-log phase liquid cultures on Middlebrook 7H11 solid medium supplemented with ADC and increasing isoxyl concentrations (indicated in μg/ml). Plates were incubated at 37°C for 4 weeks. Results are shown for two independent clones harboring pVV16 ::*PFE0555w-C*. Overexpression of *PFE0555w-C* increased *M. bovis* BCG resistance to isoxyl, suggesting that the enzyme encoded by PFE0555w is very likely to be a target of the drug in mycobacteria. In a previous study, Phetsuksiri et al. [4] demonstrated that the stearoyl-CoA DesA3 is inhibited by isoxyl. Together, these data provide further evidence that PFE0555w represents the plasmodial stearoyl-CoA desaturase.
