# Supplementary material for: Oleic Acid Biosynthesis in Plasmodium falciparum: Characterization of the Stearoyl-CoA Desaturase and Investigation as a Potential Therapeutic Target
Source: PLoS One. 2009 Sep 3;4(9):e6889. doi: 10.1371/journal.pone.0006889 (PMC2731242; doi:10.1371/journal.pone.0006889)
Supplement: Methods S1 — Preparation of cyclopropenes. (0.10 MB DOC) [file pone.0006889.s004.doc]

**Methods S1. Preparation of cyclopropenes.**

Methyl sterculate (**7**, n = 7) was prepared as described before [5]. Methyl malvalate (**7**, n = 6) and 9-(2-octylcycloprop-1-enyl)nonanoic acid methyl ester (**7**, n = 8) were prepared using slight modifications of methods that have already been published, involving the trapping of an *in situ* prepared lithiocyclopropene (**2**) by a 1,n-bromochloride (**3**) [5,6]. The racemic alcohol (8, R = H) was prepared in a similar way by trapping of the lithiocyclopropene [6] with an n-halo-aldehyde and replacement of the bromine by cyanide, followed by hydrolysis. Simple functional group conversions gave (**8**, R = Me) and (**8**, R = SiMe_3_).

**1-(6-Chlorohexyl)-2-octylcyclopropene**

*n*-Butyl lithium (1.6 M, 36.7 mmol, 22.9 ml) was added dropwise to a stirred solution of 1,1,2-tribromo-2-octylcyclopropane (5.7 g, 14.8 mmol) in dry ether (50 ml) under N_2_ at -78°C. The mixture was allowed to reach room temperature, stirred for 30 minutes and then cooled to 0°C. Hexamethylphosphoramide (5.22 g, 29.2 mmol, 5.1 ml) was added dropwise, followed by 1-chloro-6-iodo-hexane (3.28 g, 13.3 mmol). The mixture was stirred overnight at room temperature and then cooled to 0°C. Water (50 ml) was then added, the product was extracted with ether (2 x 100 ml), and the combined organic layers were washed with water (2 x 20 ml), dried and evaporated. This produced a brown oil that was purified by column chromatography (petrol) to yield pure 1-(6-chlorohexyl)-2-octylcyclopropene (1.41 g, 5.2 mmol, 39 %) as a colorless oil; ν_max_(film)/cm^-1^ 2927, 2856, 1874 and 1462; *δ*_H_ (500 MHz; CDCl_3_; Me_4_Si) 0.70 (2H, s, C*H*_2_ in ring), 0.81 (3H, t, *J* 6.9, C*H*_3_CH_2_), 1.22 (12H, m, 6 x C*H*_2_), 1.38 (2H, m, C*H*_2_), 1.48 (4H, m, 2 x C*H*_2_), 1.71 (2H, p, *J* 7.1, CH_2_C*H*_2_CH_2_Cl), 2.31 (4H, app 2t, CH_2_C*H*_2_C=CC*H*_2_CH_2_) and 3.46 (2H, t, *J* 6.8, CH_2_C*H*_2_Cl); *δ*_C_(125 MHz; CDCl_3_; Me_4_Si) 7.4, 14.1, 22.3, 26.0, 26.7, 27.2, 27.4, 28.3, 28.6, 29.3, 29.4, 31.9, 32.6, 45.1, 109.1 and 109.6. The data for this compound corresponded to those for the compound when prepared by an alternative route [6].

**7-(2-Octylcycloprop-1-enyl)-heptanenitrile**

Sodium cyanide (0.64 g, 13.1 mmol) was stirred at 60°C for 6 hr with 1-(6-chlorohexyl)-2-octyl-cyclopropene (1.18 g, 4.3 mmol) in DMSO (20 ml). Water (15 ml) was added to the cooled solution and the product was extracted with ether (2 x 100 ml). The combined organic layers were washed with saturated aqueous ammonium chloride (30 ml), dried and evaporated to give crude product. Column chromatography (petrol/ether, 5:1) yielded 7-(2-octylcycloprop-1-enyl)-heptanenitrile (5, n = 6) (0.87 g, 3.3 mmol, 78%) as a yellow oil. This showed ν_max_(film)/cm^-1^ 2927, 2856, 2246, 1871 and 1463; *δ*_H_ (500 MHz; CDCl_3_; Me_4_Si) 0.79 (2H, s, C*H*_2_ in ring), 0.90 (3H, t, *J* 6.9, C*H*_3_CH_2_), 1.31 (10H, m, 5 x C*H*_2_), 1.39 (2H, m, C*H*_2_), 1.55 (6H, m, 3 x C*H*_2_), 1.69 (2H, p, *J* 7.4, CH_2_C*H*_2_CH_2_), 2.35 (2H, t, *J* 7.1, CH_2_C*H*_2_CN) and 2.40 (4H, app 2t, CH_2_C*H*_2_C=CC*H*_2_CH_2_); *δ*_C_(125 MHz; CDCl_3_; Me_4_Si) 7.4, 14.1, 17.1, 22.3, 25.3, 25.8, 26.0, 27.0, 27.4, 28.5, 29.3, 29.4, 29.4, 31.9, 108.9, 109.8 and 119.8; m/*z* (EI) 262.2533 (M^+^). The data for this compound corresponded to those obtained for the compound when prepared by an alternative route [6].

**Methyl Malvalate (7-(2-octyl-cycloprop-1-enyl)heptanoic acid methyl ester)**

(a) Iodine (0.83 g, 3.3 mmol) in dry ether (100 g) was added dropwise to a stirred solution of 7-(2-octylcycloprop-1-enyl)heptanenitrile (0.74 g, 2.8 mmol) in dry ether (100 ml) at -90°C. The mixture was allowed to reach room temperature for 1 hour. Once the TLC showed that no starting material remained, the reaction was quenched with a saturated aqueous solution of sodium thiosulfite (30 ml) to remove any excess iodine and the aqueous layer was extracted with ether (2 x 50 ml). The combined organic layers were washed with water (2 x 20 ml), dried and evaporated to give crude product. This was purified by column chromatography (petrol/ether, 5:1) to yield 7-(1,2-diiodo-2-octylcyclopropyl)heptanenitrile (1.14 g, 2.2 mmol, 78%) as a yellow oil. This showed ν_max_(film)/cm^-1^ 2927, 2854, 2246 and 1462; *δ*_H_ (500 MHz; CDCl_3_; Me_4_Si) 0.91 (3H, t, *J* 6.9, C*H*_3_CH_2_), 1.35 (12H, m, 6 x C*H*_2_), 1.45 (2H, m, C*H*_2_), 1.57 (4H, m, 2 x C*H*_2_), 1.73 (6H, m, 3 x C*H*_2_), 2.14 (2H, m, C*H*_2_) and 2.38 (2H, t, J 7.1, CH_2_C*H*_2_CN); *δ*_C_(125 MHz; CDCl_3_; Me_4_Si) 14.1, 17.1, 22.7, 23.9, 24.3, 25.3, 27.9, 28.6, 28.8, 29.3, 29.4, 29.5, 29.8, ,31.9, 36.1, 50.5, 50.7 and 119.8; m/*z* (EI) 538.0416 (M^+^ + Na) (calculated for M^+^ + Na: 538.0448).

(b) Gaseous HCl was bubbled through a solution of 2:1 dry ether/methanol (30 ml) and the solution was cooled to -79°C, then a solution of 7-(1,2-diiodo-2-octylcyclopropyl)heptanenitrile (0.96 g, 1.9 mmol) in dry ether (20 ml) was added to the mixture. The solution was allowed to warm to room temperature and stirred overnight. A saturated solution of aqueous sodium bicarbonate (100 ml) was added slowly to this solution. The solution was neutralized to pH 7 and extracted with ether (3 x 100 ml). The combined organic layers were dried and evaporated to give a brown oil that was purified by column chromatography (petrol/ether, 5:1) to give 7-(1,2-diiodo-2-octylcyclopropyl)heptanoic acid methyl ester (0.46 g, 0.84 mmol, 45%) as a yellow oil. This showed ν_max_(film)/cm^-1^ 2927, 2854, 1741 and 1462; *δ*_H_ (500 MHz; CDCl_3_; Me_4_Si) 0.82 (3H, t, J 6.9, C*H*_3_CH_2_), 1.26 (16H, m, 8 x C*H*_2_), 1.47 (2H, m, C*H*_2_), 1.59 (4H, m, 2 x C*H*_2_), 1.69 (2H, m, C*H*_2_), 2.02 (2H, m, C*H*_2_), 2.25 (2H, t, J 7.6, CH_2_C*H*_2_CO) and 3.60 (3H, s, C*H*_3_COO); *δ*_C_(125 MHz; CDCl_3_; Me_4_Si) 14.1, 22.7, 24.2, 24.4, 24.9, 28.4, 28.8, 19.1, 29.3, 29.5, 29.6, 29.8, 31.9, 34.1, 36.1, 50.6, 50.7, 51.5 and 174.3; m/*z* (EI) 571.0514 (M^+^ + Na) (calculated for M^+^ + Na: 571.0550).

(c) *n*-Butyl lithium (1.6 M, 0.49 ml, 0.78 mmol) was added dropwise to a stirred solution of 7-(1,2-diiodo-2-octyl-cyclopropyl)-heptanoic acid methyl ester (0.39 g, 0.71 mmol) in dry ether (10 ml) at -90°C. The temperature was allowed to reach -70°C and water (1 ml) was added dropwise. The product was extracted with ether (2 x 20 ml) and the combined organic layers were washed with water (10 ml), dried and evaporated. Column chromatography (petrol/ether, 5:1) gave methyl malvalate (7, n = 6) (0.15 g, 0.51 mmol, 72%) as a yellow oil, which showed ν_max_(film)/cm^-1^ 2926, 2856, 1871, 1743 and 1462; *δ*_H_ (500 MHz; CDCl_3_; Me_4_Si) 0.69 (2H, s, C*H*_2_ in ring), 0.81 (3H, t, J 6.9, C*H*_3_CH_2_), 1.22 (14H, m, 7 x C*H*_2_), 1.47 (4H, m, 2 x C*H*_2_), 1.56 (2H, m, C*H*_2_), 2.24 (2H, t, *J* 7.6, CH_2_C*H*_2_COO), 2.30 (4H, app 2t, CH_2_C*H*_2_C=CC*H*_2_CH_2_) and 3.60 (3H, s, C*H*_3_COO); *δ*_C_(125 MHz; CDCl_3_; Me_4_Si) 7.4, 14.1, 22.7, 24.9, 25.9, 26.0, 27.2, 27.4, 28.9, 29.0, 29.3, 29.4, 31.9, 34.0, 51.4, 109.1, 109.5, 174.3; m/z 317.2442 (calculated for M^+^ + Na: 317.2457).

**1-(8-Chlorooctyl)-2-octylcyclopropene**

*n*-Butyl lithium (1.6M, 35.2 mmol, 22.1 ml) was added dropwise to a stirred solution of 1,1,2-tribromo-2-octyl cyclopropane (5.5 g, 14.2 mmol) in dry ether (50 ml) under N_2_ at -78°C. The mixture was allowed to reach room temperature, stirred for 30 minutes and then cooled to 0°C. Hexamethylphosphoramide (28.4 mmol, 5.08 g, 4.94 ml) was then added dropwise followed by 1-chloro-8-iodo-octane (3.5 g, 12.8 mmol). The mixture was stirred overnight at room temperature, cooled to 0°C, and water (50 ml) was added. The product was extracted with ether (2 x 100 ml) and the combined organic layers were washed with water (2 x 20 ml), dried and evaporated to give a brown oil. This was purified by column chromatography (petrol) to give pure 1-(8-chlorooctyl)-2-octylcyclopropene (1.81 g, 6.06 mmol, 43%) as a colorless oil. This showed ν_max_(film)/cm^-1^ 2927, 2856, 1872 and 1464; *δ*_H_ (500 MHz; CDCl_3_; Me_4_Si) 0.79 (2H, s, C*H*_2_ in ring), 0.91 (3H, t, *J* 6.9, C*H*_3_CH_2_), 1.34 (16H, m, 8 x C*H*_2_), 1.45 (2H, m, C*H*_2_), 1.57 (4H, m, 2 x C*H*_2_), 1.79 (2H, p, *J* 7.1, CH_2_C*H*_2_CH_2_Cl), 2.40 (4H, app 2t, *J* 7.1, CH_2_C*H*_2_C=CC*H*_2_CH_2_) and 3.46 (2H, t, *J* 6.8, CH_2_C*H*_2_Cl); *δ*_C_(125 MHz; CDCl_3_; Me_4_Si) 7.4, 14.1, 22.7, 26.0, 26.1, 26.9, 27.3, 27.4, 28.9, 29.2, 29.3, 29.3, 29.4, 31.9, 32.7, 45.1, 109.2 and 109.4.

**9-(2-Octyl-cycloprop-1-enyl)-nonanenitrile**

Sodium cyanide (0.81 g, 16.6 mmol) was added to a stirred solution of 1-(8-chloro-octyl)-2-octyl-cyclopropene (1.65 g, 5.5 mmol) in DMSO (25 ml). The mixture was heated to 60°C for 6 hr or until the TLC showed no starting material remaining. Water (15 ml) was added to the cooled solution and the product extracted with ether (2 x 100 ml). The combined organic layers were washed with saturated aqueous ammonium chloride (30 ml), dried and evaporated; column chromatography (petrol/ether, 5:1) gave 9-(2-octylcycloprop-1-enyl)nonanenitrile (5, n = 8) (1.16 g, 4.0 mmol, 73%) as a yellow oil. This showed ν_max_(film)/cm^-1^ 2928, 2854, 2247, 1872 and 1462; *δ*_H_ (500 MHz; CDCl_3_; Me_4_Si) 0.79 (2H, s, C*H*_2_ in ring), 0.90 (3H, t, *J* 6.7, C*H*_3_CH_2_), 1.32 (16H, m, 8 x C*H*_2_), 1.47 (2H, m, C*H*_2_), 1.57 (4H, m, 2 x C*H*_2_), 1.68 (2H, p, *J* 7.4, CH_2_C*H*_2_CH_2_CN), 2.36 (2H, t, *J* 7.1 CH_2_C*H*_2_CN) and 2.40 (4H, app 2t, J 7.1, CH_2_C*H*_2_C=CC*H*_2_CH_2_); *δ*_C_(125 MHz; CDCl_3_; Me_4_Si) 7.4, 14.1, 17.1, 22.7, 25.4, 26.0, 26.1, 27.3, 27.4, 28.7, 28.8, 29.1, 29.2, 29.3, 29.4, 31.9, 109.2, 109.5 and 119.8; m/*z* (EI) 290.2842 (M^+^) (calculated: 290.2842).

**9-(2-Octylcycloprop-1-enyl)nonanoic acid methyl ester (7, n = 8) (EH57)**

(a) Iodine (1.0 g, 3.9 mmol) in dry ether (150 ml) was added dropwise to a stirred solution of 9-(2-octyl-cycloprop-1-enyl)-nonanenitrile (0.99 g, 3.4 mmol) in dry ether (100 ml) at -90°C. The mixture was allowed to reach room temperature for 1 hr. Once the TLC showed that no starting material remained, the reaction was quenched with saturated aqueous sodium thiosulfite (50 ml) to remove any excess iodine, and the aqueous layer extracted with ether (2 x 100 ml). The combined organic layers were washed with water (2 x 25 ml), dried and evaporated. Column chromatography (petrol/ether, 5:1) gave 9-(1,2-diiodo-2-octylcyclopropyl)nonanenitrile (1.53 g, 2.8 mmol, 83%) as an orange oil. This showed ν_max_(film)/cm^-1^ 2928, 2854, 2246 and 1463; *δ*_H_ (500 MHz; CDCl_3_; Me_4_Si) 0.91 (3H, t, J 6.8, C*H*_3_CH_2_), 1.35 (18H, m, 9 x C*H*_2_), 1.53 (4H, m, 2 x C*H*_2_), 1.69 (4H, m, 2 x C*H*_2_), 1.78 (2H, m, C*H*_2_), 2.11 (2H, m, C*H*_2_), and 2.36 (2H, t, *J* 7.1 CH_2_C*H*_2_CN); *δ*_C_(125 MHz; CDCl_3_; Me_4_Si) 14.1, 17.2, 22.6, 22.7, 24.3, 24.5, 25.4, 28.6, 28.7, 28.8, 29.2, 29.3, 29.5, 29.7, 29.8, 31.9, 36.1, 50.6, 50.7 and 119.8; m/z (EI) 566.0747 (M^+^ + Na) (calculated: 566.0761).

(b) Gaseous HCl was bubbled through a solution of 2:1 dry ether/methanol (30 ml) and the solution was cooled to -79°C. A solution of 9-(1,2-diiodo-2-octylcyclopropyl)nonanenitrile (1.31 g, 2.4 mmol) in dry ether (20 ml) was added to the mixture. The solution was allowed to warm to room temperature and stirred overnight. A saturated solution of aqueous sodium bicarbonate (100 ml) was slowly added. The solution was neutralized to pH 7 and extracted with ether (3 x 100 ml). The combined organic layers were dried and evaporated to give a brown oil that was purified by column chromatography (petrol/ether, 5:1) to give 9-(1,2-diiodo-2-octylcyclopropyl)nonanoic acid methyl ester (0.85 g, 1.5 mmol, 61%) as a yellow oil. This showed ν_max_(film)/cm^-1^ 2927, 2854, 1714 and 1462; *δ*_H_ (500 MHz; CDCl_3_; Me_4_Si); 0.91 (3H, t, J 6.8, C*H*_3_CH_2_), 1.33 (20H, m, 10 x C*H*_2_), 1.65 (6H, m, 3 x C*H*_2_), 1.78 (2H, m, C*H*_2_), 2.11 (2H, m, C*H*_2_), 2.32 (2H, t, J 7.6, CH_2_C*H*_2_COO) and 3.69 (3H, s, COOC*H*_3_); *δ*_C_(125 MHz; CDCl_3_; Me_4_Si) 14.1, 22.6, 22.7, 24.4, 24.5, 25.0, 28.7, 28.8, 29.2, 29.3, 29.4, 29.5, 29.7, 29.8, 31.9, 34.1, 36.1, 50.7, 50.7, 51.5 and 174.3; m/z (EI) 599.0826 (M^+^ + Na) (calculated: 599.0863).

(c) *n*-Butyl lithium (1.6 M, 1.3 ml, 2.1 mmol) was added dropwise to a stirred solution of 9-(1,2-diiodo-2-octylcyclopropyl)nonanoic acid methyl ester (0.61 g, 1.9 mmol) in dry ether (10 ml) at -90°C. The temperature was allowed to reach -70°C and water (1 ml) was added dropwise. The product was extracted with ether (2 x 20 ml) and the combined organic layers were washed with water (10 ml), dried and evaporated. Column chromatography (petrol/ether, 5:1) gave 9-(2-octylcycloprop-1-enyl)nonanoic acid methyl ester (7, n = 8) (0.18 g, 0.56 mmol, 29%) as a colorless oil. This showed ν_max_(film)/cm^-1^ 2926, 2854, 1869, 1744 and 1464; *δ*_H_ (500 MHz; CDCl_3_; Me_4_Si) 0.79 (2H, s, C*H*_2_ in ring), 0.91 (3H, t, *J* 7.0, C*H*_3_CH_2_), 1.32 (18H, m, 9 x C*H*_2_), 1.56 (4H, m, 2 x C*H*_2_), 1.64 (2H, m, C*H*_2_), 2.32 (2H, t, J 7.6, CH_2_C*H*_2_COO), 2.39 (4H, app 2t, CH_2_C*H*_2_C=CC*H*_2_CH_2_) and 3.69 (3H, s, COOC*H*_3_); *δ*_C_(125 MHz; CDCl_3_; Me_4_Si) 7.4, 14.1, 22.7, 25.0, 26.0, 26.1, 27.3, 27.4, 29.1, 29.2, 29.2, 29.3, 29.3, 29.4, 31.9, 34.1, 51.4, 109.3, 109.4 and 174.3; m/z (EI) 345.2752 (M^+^ + Na) (calculated: 345.2770).

**(±)-7-Bromo-1-(2-octylcycloprop-1-enyl)heptan-1-ol**

*n*-Butyl lithium (1.6 M, 48.9 mmol, 30.58 ml) was added dropwise to a stirred solution of 1,1,2-tribromo-2-octyl cyclopropane (7.65 g, 19.6 mmol) in dry ether (60 ml) under N_2_ at -78°C. The mixture was allowed to reach room temperature, stirred for 30 minutes and then cooled to 0°C. Hexamethylphosphoramide (39.1 mmol, 7.0 g, 6.8 ml) was added dropwise followed by 7-bromo-heptanal (3.4 g, 17.6 mmol). The mixture was stirred overnight at room temperature, cooled to 0°C and water (50 ml) was added. The product was extracted with ether (2 x 100 ml) and the combined organic layers were washed with water (2 x 20 ml), dried and evaporated. Column chromatography (petrol/ether, 5:1) gave pure 7-bromo-1-(2-octylcycloprop-1-enyl)heptan-1-ol (4.03 g, 11.7 mmol, 66 %) as a colorless oil. This showed ν_max_(film)/cm^-1^ 3355 (br), 2928, 2856, 1868 and 1463; *δ*_H_ (500 MHz; CDCl_3_; Me_4_Si) 0.90 (3H, t, *J* 6.7, C*H*_3_CH_2_), 0.96 (2H, s, C*H*_2_ in ring), 1.40 (16H, m, 8 x C*H*_2_), 1.58 (2H, m, C*H*_2_), 1.72 (3H, m, C*H*_2_and O*H*), 1.88 (2H, p, *J* 6.9, CH_2_C*H*_2_CH_2_), 2.44 (2H, dt, *J* 0.9 and 6.6, CH_2_C*H*_2_CCH(H)), 3.42 (2H, t, *J* 6.7, CH_2_C*H*_2_Br) and 4.63 (1H, t, *J* 6.3, OHC*H*CH_2_); *δ*_C_(125 MHz; CDCl_3_; Me_4_Si) 7.5, 14.1, 22.7, 25.0, 25.9, 27.4, 28.1, 28.7, 29.3, 29.4, 29.4, 31.9, 32.7, 33.9, 35.6, 67.9, 110.4 and 112.6.

**(±)-8-Hydroxy-8-(2-octylcycloprop-1-enyl)octanenitrile**

Sodium cyanide (1.28 g, 26.1 mmol) was added to a stirred solution of 7-bromo-1-(2-octyl-cycloprop-1-enyl)-heptan-1-ol (3.0 g, 8.7 mmol) in DMSO (40 ml). The mixture was heated to 60°C for 3 hr or until the TLC showed that no starting material remained. Water (15 ml) was added to the cooled solution and the product was extracted with ether (2 x 100 ml). The combined organic layers were washed with saturated aqueous ammonium chloride (30 ml), dried and evaporated. Column chromatography (petrol/ether, 1:1) gave 8-hydroxy-8-(2-octyl-cycloprop-1-enyl)-octanenitrile (1.78 g, 6.1 mmol, 70 %) as a yellow oil. This showed ν_max_(film)/cm^-1^ 3441 (br), 2928, 2857, 2247, 1869 and 1465; *δ*_H_ (500 MHz; CDCl_3_; Me_4_Si); 0.89 (3H, t, *J* 6.9, C*H*_3_CH_2_), 0.96 (2H, s, C*H*_2_ in ring), 1.41 (16H, m, 8 x C*H*_2_), 1.58 (2H, m, C*H*_2_), 1.70 (5H, m, 2 x C*H*_2_ and O*H*), 2.35 (2H, t, *J* 7.2, CH_2_C*H*_2_CN), 2.46 (2H, dt, *J* 0.95 and 7.2, CH_2_C*H*_2_CCH(H)) and 4.62 (1H, t, *J* 6.3, OHC*H*CH_2_); *δ*_C_(125 MHz; CDCl_3_; Me_4_Si) 7.5, 14.1, 17.1, 22.7, 24.9, 25.3, 25.9, 27.4, 28.6, 28.7, 29.2, 29.3, 29.4, 31.9, 35.6, 67.8, 110.4, 112.7 and 119.7; m/z (EI) 314.2460 (M^+^ + Na) (calculated: 314.2460).

**(±)-8-Hydroxy-8-(2-octylcycloprop-1-enyl)-octanoic acid methyl ester (EH164)**

Sodium hydroxide (0.88 g, 21.9 mmol) was dissolved in a mixture of ethanol (7 ml) and water (1 ml). 8-Hydroxy-8-(2-octylcycloprop-1-enyl)-octanenitrile (1.1 g, 3.78 mmol) was then added and the mixture was heated under reflux under nitrogen for 8 hr. The ethanol was evaporated and a 0.1 M tetrabutylammonium hydroxide solution (80 ml) was added to the residue. Dichloromethane (80 ml) and iodomethane (2.36 ml, 37.8 mmol, 5.37 g) were then added and the mixture was stirred for 12 hr. The reaction mixture was separated and the dichloromethane layer was washed with water (3 x 30 ml) and brine (30 ml). The solution was dried, filtered and evaporated to yield a crude product. This was purified by column chromatography (petrol/ether, 6:4) to give 8-hydroxy-8-(2-octyl-cycloprop-1-enyl)-octanoic acid methyl ester (0.81 g, 2.5 mmol, 66 %) as a yellow oil. ν_max_(film)/cm^-1^ 1742, 1869, 2857, 2928 and 3430 (br); *δ*_H_ (500 MHz; CDCl_3_; Me_4_Si) 0.89 (3H, t, *J* 6.9, C*H*_3_CH_2_), 0.94 (2H, s, C*H*_2_ in ring), 1.35 (16H, m, 8 x C*H*_2_), 1.63 (6H, m, 3 x C*H*_2_), 1.78 (1H, br s, O*H*), 2.31 (2H, t, *J* 7.5, CH_2_C*H*_2_C=C), 2.44 (2H, dt, *J* 0.95 and 7.2, C*H*_2_CHOH), 3.67 (3H, s, COOC*H*_3_) and 4.61 (1H, t, *J* 6.4, CH_2_C*H*OH); *δ*_C_(125 MHz; CDCl_3_; Me_4_Si) 7.5, 14.1, 22.7, 24.9, 25.0, 25.9, 27.4, 29.1, 29.1, 29.3, 29.3, 29.4, 31.9, 34.1, 35.7, 51.4, 67.9, 110.4, 112.5 and 174.3.

**(±)-8-Acetoxy-8-(2-octylcycloprop-1-enyl)octanoic acid methyl ester (EH172)**

Pyridine (0.24 g, 3.08 mmol, 0.25 ml) and acetic anhydride (0.31 g, 3.08 mmol, 0.29 ml) were added to a stirred solution of 8-hydroxy-8-(2-octyl-cycloprop-1-enyl)-octanoic acid methyl ester (0.2 g, 0.617 mmol) in dry toluene (10 ml) at room temperature under N_2_. The mixture was stirred overnight. The solvent was then evaporated and dilute acid was added to the residue. The solution was then extracted with dichloromethane (3 x 20 ml), dried and evaporated to yield a crude oil. Column chromatography (petrol/ether, 7:3) gave 8-acetoxy-8-(2-octylcycloprop-1-enyl)octanoic acid methyl ester (0.22 g, 0.601 mmol, 97 %) as a yellow oil. This showed ν_max_(film)/cm^-1^ 2928, 2856, 1873 and 1742; *δ*_H_ (500 MHz; CDCl_3_; Me_4_Si) 0.88 (3H, t, *J* 6.9, C*H*_3_CH_2_), 0.95 (1H, d, J 8.5, C*H*(H) in ring), 0.96 (1H, d, *J* 8.7, C*H*(H) in ring), 1.28 (16H, m, C*H*_2_), 1.54 (2H, m, C*H*_2_), 1.61 (2H, m, C*H*_2_), 1.75 (2H, m, C*H*_2_), 2.07 (3H, s, CHCOOC*H*_3_), 2.30 (2H, t, *J* 7.6, CH_2_C*H*_2_C=C), 2.40 (2H, dt, *J* 1.25 and 7.25, AcOCHC*H*_2_CH_2_), 3.66 (3H, s, COOC*H*_3_) and 5.66 (1H, t, *J* 6.5, AcOC*H*CH_2_); *δ*_C_(125 MHz; CDCl_3_; Me_4_Si) 8.4, 14.1, 21.1, 22.6, 24.8, 24.9, 25.9, 27.2, 29.0, 29.2, 29.3, 31.8, 32.6, 34.0, 51.4, 70.3, 108.0, 114.2, 170.4 and 174.1.

**(±)-8-(*tert*-butyldimethylsilanyloxy)-8-(2-octylcycloprop-1-enyl)octanoic acid methyl ester (EH171)**

Imidazole (0.10 g, 1.5 mmol) was added to a stirred solution of 8-hydroxy-8-(2-octyl-cycloprop-1-enyl)-octanoic acid methyl ester (0.2 g, 0.617 mmol) in dry DMF (10 ml) at room temperature. The solution was cooled to 0°C and tert-butyldimethylchlorosilane (0.12 g, 0.80 mmol) was added. The cooling bath was removed and the reaction mixture was stirred at 45°C for 20 hr. A few crystals of 4-dimethylaminopyridine, imidazole (0.10 g, 1.5 mmol) and tert-butyldimethylchlorosilane (0.12 g, 0.80 mmol) were added to the solution and the temperature was increased to 70°C. The reaction mixture was then stirred for another 20 hr. Once TLC showed complete reaction, the DMF was removed by flash distillation. Water (30 ml) was added and the product was extracted with dichloromethane (3 x 30 ml). The combined organic layers were washed with water (25 ml), dried and evaporated to give a crude product. This was purified by column chromatography (petrol/ether, 7:3) to give 8-(*tert*-butyldimethylsilanyloxy)-8-(2-octylcycloprop-1-enyl)octanoic acid methyl ester (0.17 g, 0.387 mmol, 63 %) as a yellow oil. This showed ν_max_(film)/cm^-1^ 2929, 2856, 1870 and 1744; *δ*_H_ (500 MHz; CDCl_3_; Me_4_Si) 0.00 (3H, s, C*H*_3_Si), 0.02 (3H, s, C*H*_3_Si), 0.87 (14H, m), 1.26 (16H, m, 8 x C*H*_2_), 1.51 (2H, m, C*H*_2_), 1.59 (4H, m, C*H*_2_), 2.26 (2H, t, *J* 7.5, CH_2_C*H*_2_C=C), 2.36 (2H, t, *J* 7.1, SiOCHC*H*_2_CH_2_), 3.62 (3H, s, COOC*H*_3_), 4.56 (1H, t, *J* 6.3, CH_2_CHOSi); *δ*_C_(125 MHz; CDCl_3_; Me_4_Si) -5.1, -4.6, 8.0, 14.1, 18.3, 22.7, 24.9, 25.1, 25.8, 25.8, 27.2, 29.1, 29.2, 29.3, 29.4, 29.4, 31.9, 34.0, 36.2, 51.3, 68.5, 111.1, 111.3 and 174.2.

**(±)-1-(7-Bromo-1-methoxyheptyl)-2-octylcyclopropene**

*n*-Butyl lithium (1.6 M, 43.2 mmol, 26.9 ml) was added dropwise to a stirred solution of 1,1,2-tribromo-2-octylcyclopropane (6.75 g, 17.2 mmol) in dry ether (60 ml) under N_2_ at -78°C. The mixture was allowed to reach room temperature, stirred for 30 minutes and then cooled to 0°C. Hexamethylphosphoramide (34.5 mmol, 6.18 g, 6.0 ml) was then added dropwise, followed by 7-bromo-heptanal (3.0 g, 15.5 mmol). The mixture was stirred for 3 hr and iodomethane (172.7 mmol, 24.51 g, 10.76 ml) was added. The mixture was stirred overnight at room temperature and then cooled to 0°C and water (70 ml) was added. The product was extracted with ether (2 x 100 ml) and the combined organic layers washed with water (2 x 20 ml), dried and evaporated to give crude product as a brown oil. Column chromatography (petrol/ether, 5:1) gave pure 1-(7-bromo-1-methoxyheptyl)-2-octylcyclopropene (3.24 g, 9.0 mmol, 53%) as a colorless oil. This showed ν_max_(film)/cm^-1^ 2925, 2854, 1866 and 1463; *δ*_H_ (500 MHz; CDCl_3_; Me_4_Si) ; 0.90 (3H, t, *J* 6.9, C*H*_3_CH_2_), 0.94 (1H, d, *J* 8.5, C*H*(H) in ring), 0.97 (1H, d, *J* 8.5, C*H*(H) in ring), 1.38 (16 H, m, 8 x C*H*_2_), 1.59 (2H, p, *J* 7.3, CH_2_C*H*_2_CH_2_), 1.69 (2H, m, C*H*_2_), 1.81 (2H, m, C*H*_2_), 2.46 (2H, dt, J 0.6 and 7.3, CH_2_C*H*_2_CCH(H)), 3.19 (2H, t, *J* 7.0, CH_2_C*H*_2_Br), 3.33 (3H, s, C*H*_3_O) and 4.21 (1H, t, *J* 6.3, CH_3_OC*H*CH_2_); *δ*_C_(125 MHz; CDCl_3_; Me_4_Si) 7.0, 7.5, 14.1, 22.7, 25.1, 26.1, 27.2, 28.5, 29.2, 29.3, 29.4, 30.4, 31.9, 33.3, 33.4, 26.6, 76.3, 108.6 and 114.1.

**(±)-8-Methoxy-8-(2-octylcycloprop-1-enyl)-octanenitrile**

Sodium cyanide (0.97 g, 19.8 mmol) was added to a stirred solution of 1-(7-bromo-1-methoxy-heptyl)-2-octylcyclopropene (2.97 g, 8.27 mmol) in DMSO (40 ml). The mixture was heated to 60°C for 3 hr or until the TLC showed that no starting material remained. Water (15 ml) was added to the cooled solution and the product was extracted with ether (2 x 100 ml). The combined organic layers were washed with saturated aqueous ammonium chloride (30 ml), dried and evaporated. Column chromatography (petrol/ether, 5:2) gave pure 8-methoxy-8-(2-octylcycloprop-1-enyl)octanenitrile (1.85 g, 6.1 mmol, 73%) as a yellow oil. This showed ν_max_(film)/cm^-1^ 2925, 2854, 2246, 1865 and 1465; *δ*_H_ (500 MHz; CDCl_3_; Me_4_Si) 0.91 (3H, t, *J* 6.8, C*H*_3_CH_2_), 0.95 (1H, d, *J* 8.5, C*H*(H) in ring), 0.97 (1H, d, *J* 8.2, C*H*(H) in ring), 1.35 (14H, m, 7 x C*H*_2_), 1.47 (2H, m, C*H*_2_), 1.59 (2H, m, C*H*_2_), 1.71 (4H, m, 2 x C*H*_2_), 2.35 (2H, t, *J* 7.1, CH_2_C*H*_2_CN), 2.47 (2H, t, *J* 7.3, CH_2_C*H*_2_C=C), 3.34 (3H, s, C*H*_3_O) and 4.21 (1H, t, *J* 6.6, CH_3_OC*H*CH_2_); *δ*_C_(125 MHz; CDCl_3_; Me_4_Si) 7.5, 14.1, 17.1, 22.7, 25.0, 25.3, 26.1, 27.2, 28.6, 28.7, 29.3, 39.4, 29.4, 31.8, 33.3, 56.6, 76.3, 108.6, 114.2 and 119.8; m/z (EI) 328.2595 (calculated: 328.2616).

**(±)-8-Methoxy-8-(2-octylcycloprop-1-enyl)octanoic acid methyl ester (EH87)**

Sodium hydroxide (0.39 g, 9.6 mmol) was dissolved in a mixture of ethanol (3.5 ml) and water (0.5 ml). 8-Methoxy-8-(2-octyl-cycloprop-1-enyl)-octanenitrile (0.5 g, 1.6 mmol) was then added and the mixture heated under reflux under nitrogen for 8 hr. The ethanol was evaporated and a 0.1 M tetrabutylammonium hydroxide solution (40 ml) was added to the residue. Dichloromethane (40 ml) and iodomethane (1.02 ml, 16.4 mmol, 2.3 g) were then added and the mixture was stirred for 12 hr. The reaction mixture was separated and the organic layer was washed with water (3 x 20 ml) and brine (20 ml). The solution was dried, filtered and evaporated to yield a crude product. This was purified by column chromatography (petrol/ethyl acetate, 5:1) to give 8-methoxy-8-(2-octyl-cycloprop-1-enyl)-octanoic acid methyl ester (0.52 g, 1.5 mmol, 93%) as a colorless oil. ν_max_(film)/cm^-1^ 2927, 2855, 1863, 1743 and 1464; *δ*_H_ (500 MHz; CDCl_3_; Me_4_Si) 0.89 (3H, t, *J* 6.9, C*H*_3_CH_2_), 0.93 (1H, d, *J* 8.5, C*H*(H) in ring), 0.94 (1H, d, *J* 8.5, C*H*(H) in ring), 1.31 (16H, m, 8 x C*H*_2_), 1.65 (6H, m, 3 x C*H*_2_), 2.31 (2H, t, *J* 7.6, CH_2_C*H*_2_COO), 2.45 (2H, t, *J* 7.25, CH_2_C*H*_2_C=C), 3.32 (3H, s, OC*H*_3_), 3.67 (3H, s, COOC*H*_3_) and 4.19 (1H, t, *J* 6.5, CH_3_OC*H*CH_2_); *δ*_C_(125 MHz; CDCl_3_; Me_4_Si) 7.5, 14.1, 22.6, 24.9, 25.2, 26.1, 27.2, 29.1, 29.2, 29.3, 29.3,

29.4, 31.9, 33.3, 34.1, 51.4, 56.5, 76.4, 108.7, 114.0 and 174.2; m/z (EI) 361.2679 (M^+^ + Na) (calculated: 361.2719).

# References

1. Berenbaum MC (1978) A method for testing for synergy with any number of agents. J Infect Dis 137: 122-130.

2. Gupta S, Thapar MM, Wernsdorfer WH, Bjorkman A (2002) In vitro interactions of artemisinin with atovaquone, quinine, and mefloquine against Plasmodium falciparum. Antimicrob Agents Chemother 46: 1510-1515.

3. Jackson M, Crick DC, Brennan PJ (2000) Phosphatidylinositol is an essential phospholipid of mycobacteria. J Biol Chem 275: 30092-30099.

4. Phetsuksiri B, Jackson M, Scherman H, McNeil M, Besra GS, et al. (2003) Unique mechanism of action of the thiourea drug isoxyl on Mycobacterium tuberculosis. J Biol Chem 278: 53123-53130.

5. Baird MS, Dale, C.M., Lytollis, W., and Simpson, M.J (1992) Tetrahedron Letts 33: 1521-1522.

6. Baird MS, and Grehan, B (1993) J Chem Soc Perkin Trans 1: 1547-1548
